# Fc-mediated immune stimulating, pro-inflammatory and antitumor effects of anti-HER2 IgE against HER2-expressing and trastuzumab-resistant tumors

**DOI:** 10.1136/jitc-2024-010945

**Published:** 2025-03-12

**Authors:** Melanie Grandits, Lais C G F Palhares, Gabriel Osborn, Jitesh Chauhan, Katie Stoker, Heng Sheng Sow, Rebecca Adams, Alex J McCraw, Alicia Chenoweth, Sofia Vlasova, Jacobo López-Abente, Kristina M Ilieva, James Birtley, Sophia Tsoka, Elizabeth Hardaker, Kevin FitzGerald, Sophia N Karagiannis, Heather J Bax

**Affiliations:** 1 St. John’s Institute of Dermatology, School of Basic & Medical Biosciences & KHP Centre for Translational Medicine, King's College London, London, UK; 2 Epsilogen Ltd, Waterfront, ARC West London, Manbre Road, Hammersmith, London, UK; 3 Department of Informatics, Faculty of Natural, Mathematical and Engineering Sciences, King's College London, London, UK; 4 Breast Cancer Now Research Unit, School of Cancer & Pharmaceutical Sciences, King's College London, London, UK

**Keywords:** Monoclonal antibody, Solid tumor, Immunotherapy

## Abstract

**Background:**

Anti-human epidermal growth factor receptor 2 (HER2) IgG1-based antibody therapies significantly improve cancer prognosis, yet intrinsic or acquired resistance to fragment antigen-binding (Fab)-mediated direct effects commonly occurs. Most resistant tumors retain antigen expression and therefore remain potentially targetable with anti-HER2 therapies that promote immune-mediated responses. Tumor-antigen-specific IgE class antibodies can mediate powerful immune cell-mediated effects against different cancers and have been shown to activate IgE Fc receptor-expressing monocytes. We previously reported the engineering of a trastuzumab-equivalent anti-HER2 IgE antibody and showed early evidence of Fc-mediated cancer cell-targeting effects. In the present study, we evaluated the anti-tumoral functions of two anti-HER2 IgEs, trastuzumab and pertuzumab IgE.

**Methods:**

In vitro functionality of the two anti-HER2 antibodies was assessed by HER2 phosphorylation and ligand-independent viability assays, as well as basophil (RBL-SX38) degranulation, antibody-dependent cellular cytotoxicity/antibody-dependent cellular phagocytosis(ADCC/ADCP) assays and primary monocyte stimulation assays. The potential to trigger a hypersensitivity type I reaction was investigated using the basophil activation test (BAT). anti-tumoral efficacy was assessed in two humanized HER2+, trastuzumab-resistant models in vivo. Changes in the tumor microenvironment were assessed by flow cytometry or bulk RNA sequencing.

**Results:**

We demonstrate the anti-tumoral and immunostimulatory functions of two anti-HER2 IgEs derived from variable region sequences of the clinically available trastuzumab and pertuzumab IgG1 antibodies. IgE engagement of monocytes via the Fc region induced tumor cell cytotoxicity and a pro-inflammatory shift with upregulation of immune-stimulatory CD40, CD80 and CD86, and downregulation of scavenger CD163, cell surface molecules. This was accompanied by enhanced pro-inflammatory tumor necrosis factor (TNF)-α, interleukin (IL)-6, IL-1β cytokine production. The absence of basophil activation by anti-HER2 IgEs ex vivo in whole blood points to potentially safe administration in humans. In two trastuzumab-resistant HER2+ tumor xenograft models in immunodeficient mice reconstituted with human immune cells, the trastuzumab-equivalent anti-HER2 IgE restricted tumor growth. Treatment was associated with enriched classical (CD14^+^CD16^–^) monocyte and lower alternatively-activated (CD163^+^CD206^+^) macrophage infiltration, and higher densities of activated CD4^+^ (CD127^lo^CD25^hi^) T cells and favorable effector T cell(Teff) to regulatory T cell (Treg) ratios in tumors.

**Conclusion:**

Collectively, anti-HER2 IgE maintains Fab-mediated antitumor activity, induces Fc-mediated effects against HER2-expressing tumor cells, and stimulates remodeling of the immune microenvironment in tumors to promote pro-inflammatory cell phenotypes which could translate to improved outcomes for patients.

**WHAT IS ALREADY KNOWN ON THIS TOPIC**

- Resistance to fragment antigen-binding (Fab)-mediated and fragment crystallizable (Fc)-mediated effects of anti-human epidermal growth factor receptor 2 (HER2) IgG1 monoclonal antibody treatments is known to limit clinical response. Tumor-antigen-specific IgE class antibodies can harness a distinct arm of the immune system, however, these effects have not been fully elucidated against HER2-expressing cancers.

**WHAT THIS STUDY ADDS**

- We generated anti-HER2 trastuzumab-equivalent and pertuzumab-equivalent IgE antibodies and demonstrated Fc-mediated effects and pro-inflammatory immune cell switch directed against HER2-expressing cancer cells, including those resistant to trastuzumab Fab-mediated effects.

**HOW THIS STUDY MIGHT AFFECT RESEARCH, PRACTICE OR POLICY**

- IgE induces immune cell stimulation and reprogramming of the tumor microenvironment, potentially offering new therapy options for HER2-expressing, treatment-resistant cancers.

## Introduction

 Approximately 20% of breast cancers overexpress the membrane tyrosine kinase, human epidermal growth factor receptor 2 (HER2), leading to uncontrolled cell proliferation and survival through increased receptor dimerization. Therefore, HER2+ tumors associate with rapid progression and poor outcomes.^1^

Patient survival has significantly improved with anti-HER2 IgG1 monoclonal antibody (mAbs) treatments, including trastuzumab (Tras) (Herceptin) and pertuzumab (Per) (Perjeta). Tras binds to domain IV of HER2, thereby blocking mainly ligand-independent homodimerization of HER2. Per binds to domain II, blocking ligand-dependent heterodimerization of HER2 with other members of the epidermal growth factor receptor family. Blocking dimerization can interfere with downstream phosphorylation, thus reducing proliferation.^2^ These direct fragment antigen-binding (Fab)-mediated effects have largely been regarded as the main mechanism of action for anti-HER2 antibodies. Immune cell-mediated effects such as antibody-dependent cellular cytotoxicity (ADCC) have been reported, although their contribution to overall efficacy in preclinical and clinical settings remains unclear.^1^

Anti-HER2 antibodies are first-line therapy for patients with high tumor expression of HER2 (HercepTest 3+ and HercepTest 2+ with fluorescence in situ hybridization). However, intrinsic or acquired resistance through several mechanisms such as mutations downstream of the HER2 phosphorylation pathway, are well documented, making HER2 dimerization redundant for cell proliferation.^3^,^5^ Nevertheless, the majority of tumors resistant to Fab-mediated effects of anti-HER2 antibodies (termed “trastuzumab-resistant”), retain detectable cell surface levels of HER2. Thus, these may be targetable with anti-HER2 therapies featuring other modes of action, including immune-mediated mechanisms.^4^

IgG1 is currently the isotype of choice for antibody immunotherapeutic agents when Fc-mediated immune-stimulating effects are desirable.^6^ However, FcγR affinity-reducing polymorphisms and several immunomodulatory mechanisms in the tumor microenvironment (TME) including alternatively-activated immune effector cells, and expression of inhibitory Fcγ receptors, may limit the ability of IgG1 antibodies to effectively stimulate immune responses within tumor lesions.^7^,^10^ IgE class mAbs may offer an alternative approach to activate the immune microenvironment. Known properties of IgE class antibodies, such as very high affinity for cognate Fcε receptors and lack of inhibitory Fc receptors, might translate to effective engagement of tumor-resident immune cells, prolonged tissue residency, and orchestration of potent effector functions in immune privileged Th2-biased environments such as those of tumors.^11^,^13^ In recent years, IgE-based therapies have been shown to be effective in significantly reducing tumor burden in in vivo preclinical models, including melanoma and ovarian cancer.^14^,^16^ These effects are thought to feature significant recruitment and stimulation of monocytes and macrophages and re-conditioning of the TME towards pro-inflammatory, anti-tumoral states. Furthermore, encouraging results observed in a clinical trial of the first-in-class IgE antibody, MOv18 IgE, indicate tolerability, with evidence of anticancer activity in a patient with ovarian cancer.^17^ These point to IgE class antibodies as a promising novel modality for cancer therapy.

We previously reported the engineering and early functional evaluation of a Tras-equivalent anti-HER2 IgE antibody. In these studies, Tras IgE triggered degranulation of a basophilic leukemia cell line and ADCC by immune effector cells against high HER2-expressing cell lines in vitr*o*.^13 18^ Furthermore, another anti-HER2 IgE (clone C6MH3-B1) has previously been shown to enhance antigen-presentation in human dendritic cells in vitro and significantly prolong the survival of human FcεRIα transgenic mice bearing HER2+tumors. This anti-HER2 IgE has also been well-tolerated in cynomolgus monkeys.^19^ However, anti-HER2 IgE antibodies have not been evaluated in HER2+, Tras-resistant disease settings. Moreover, it is unclear whether an IgE targeting HER2-expressing cancer can drive the stimulation and pro-inflammatory repolarization of key immune effector cells, such as monocytes, or influence immunosuppressive features in the TME.

In this study, we evaluated two anti-HER2 antibodies with the variable regions of the clinically used IgG1 Tras and Per generated with human IgE Fc regions (Tras IgE and Per IgE). We tested the retention of antibody binding to HER2-expressing cancer cells, and its impact on HER2 phosphorylation and cancer cell proliferation in vitro. We assessed the in vitro activity of Tras and Per IgE to engender functional degranulation, cytotoxicity, and immune cell stimulation in co-cultures with cancer cells, including with Tras-resistant cells. We interrogated the potential of anti-HER2 IgEs to trigger early signs of type I hypersensitivity reactions in a whole blood assay (basophil activation test, BAT) as an early readout of safety. We explored the in vivo functions of Tras IgE in two murine HER2+models: a HercepTest 3+ equivalent Tras-resistant tumor in CD34+humanized mice; and a HercepTest 2+ equivalent Tras-resistant tumor in mice reconstituted with human peripheral blood mononuclear cells (PBMCs). Furthermore, we investigated effector cell infiltration and pathway activation in tumors following IgE treatment in vivo. Overall, our study investigates the immune-stimulating attributes of HER2-specific IgE for the treatment of HER2-expressing and Tras-resistant cancers.

## Materials and methods

### Cancer cell lines and effector cells

Cancer and effector cell lines were sourced and maintained as described in online supplemental methods. HER2 expression levels were determined using BD Quantibrite beads and an anti-HER2 PE-conjugated antibody (HRB2/258) (details online supplemental methods). PBMCs of healthy volunteers were isolated using Ficoll-Paque as previously described. Primary monocytes were isolated from healthy volunteer PBMCs by negative selection (Pan Monocyte Isolation Kit, Miltenyi), as previously described.^20^

### Antibody production and characterization

Tras IgE and Per IgE, as well as an isotype control not specific to HER2 (NIP IgE), were produced as previously described.^18^ Antibodies were purified using a CaptureSelect IgE column followed by a buffer exchange to phosphate-buffered saline (PBS) pH 7.4.^21^ SDS-PAGE, and cell-surface binding to hHER2 (SKBR3) and FcεRI (RBL-SX38 rat basophilic leukemia cell/whole blood basophils) were performed as described in online supplemental methods.

### In vitro and ex vivo assays

In vitro IgE antibody functions were evaluated by assays measuring HER2 phosphorylation and ligand-independent viability of HER2+cancer cells, RBL-SX38 degranulation assay, ADCC/antibody-dependent cellular phagocytosis (ADCP) assays with healthy volunteer PBMCs or U937s as effector cells, primary monocyte stimulation assays, and the BAT (details in online supplemental methods).

### In vivo model development and antibody treatment efficacy studies

Efficacy of Tras IgE was assessed in vivo using Tras-resistant SKOv33 (HER2 3+) and JIMT-1 (HER2 2+) mouse models (as described in detail in online supplemental methods).

To determine tumor-associated immune infiltrates, the SKOv3 tumors were enzymatically dissociated and assessed by flow cytometry as detailed in online supplemental methods. To assess the transcriptomic profile of JIMT-1 tumors, bulk RNA sequencing was carried out by Azenta/Genewiz and results analyzed using DESeq2 (V.1.40.2) for differentially expressed genes, gprofiler2 (V.0.2.2) for gene over-representation analysis, and Reactome (V.7.4) for Gene Set Enrichment Analysis (GSEA).

## Results

### Tras and Per IgE antibodies mediate Fab-mediated and Fc-mediated anti-tumoral functions in vitro

**Figure 1:**
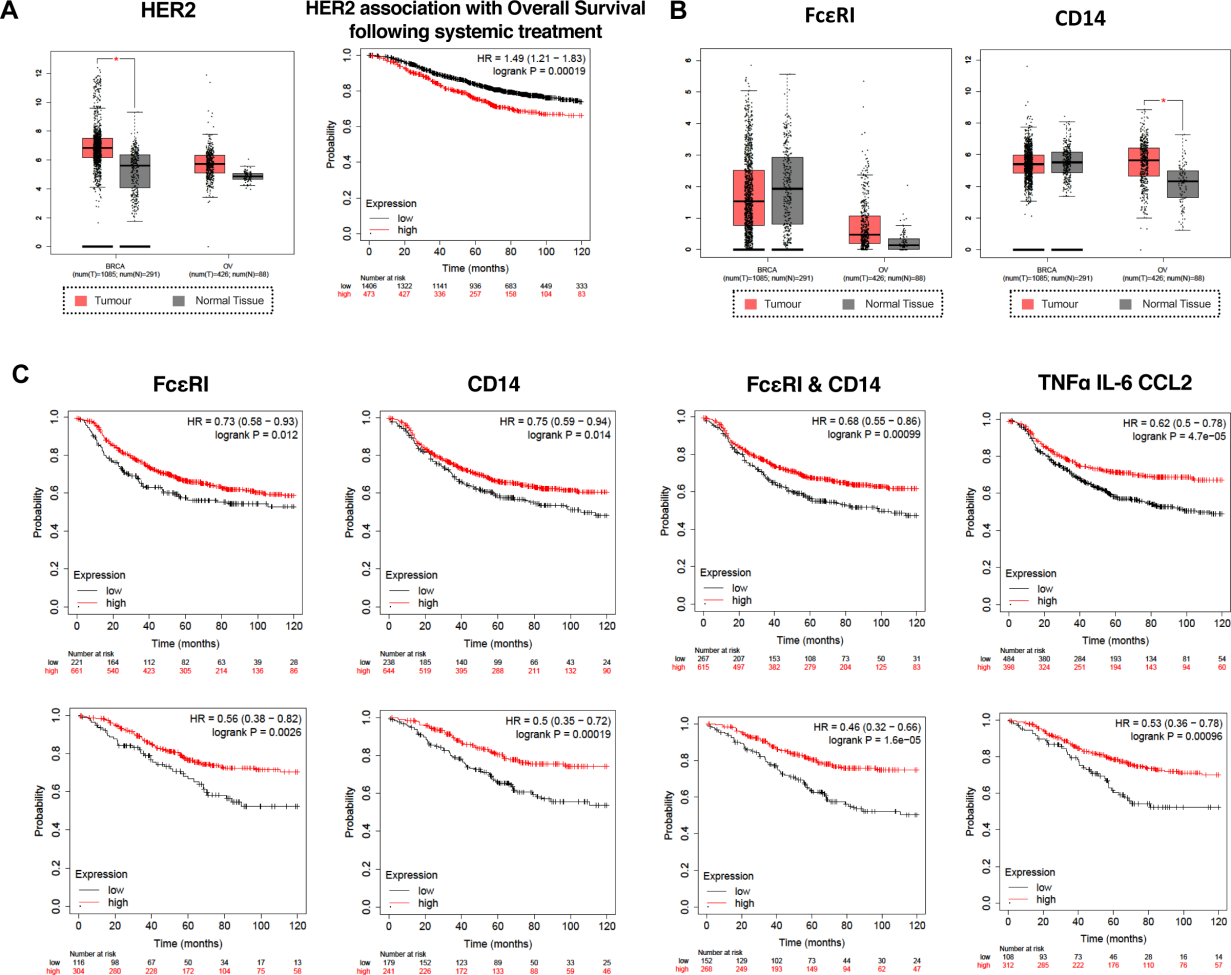
Expression of HER2, FcεRI, CD14 and pro-inflammatory cytokine markers in breast and ovarian cancer and association with survival. (**A**) Left: expression of HER2 in tumors (red) and in the equivalent normal tissues (gray). Right: Kaplan-Meier (KM) plots showing association of HER2 expression with worse overall survival following systemic treatment (endocrine therapy and chemotherapy). (**B**) Expression of FcεRI, CD14 in breast cancer and (red) and in the equivalent normal tissues (gray). (**C**) FcεRI, CD14, and pro-inflammatory cytokine levels are associated with more favorable recurrence-free and overall survival (10 years) in HER2+breast cancer. Data were obtained from the Gene Expression Profiling Interactive Analysis online database (http://gepia.cancer-pku.cn/index.html),^22^ accessed on March 29, 2024. Survival outcome analysis performed using the KM plotter online tool (https://kmplot.com/analysis/)^23^ accessed on March 29, 2024. CCL2, C-C motif chemokine ligand 2; HER2, human epidermal growth factor receptor 2; IL, interleukin; TNF, tumor necrosis factor.

To evaluate whether IgE antibodies may engage with FcεRs to engender Fc-mediated effector functions against HER2+tumors, we analyzed publicly available data sets.^22 23^ HER2 overexpression was confirmed in ovarian and breast tumors, in the latter it was associated with reduced overall survival (OS) following systemic treatment (endocrine therapy and chemotherapy) (figure 1A). Both, FcεRI and the monocyte marker CD14, were expressed in HER2+ breast and ovarian cancers (figure 1B). Higher levels of expression of FcεRI, or CD14, or of both combined, associated with better recurrence-free survival (RFS) and OS (10 years) in HER2+ breast cancer (figure 1C). Additionally, combined higher expression of pro-inflammatory cytokines tumor necrosis factor (TNF)-α, interleukin (IL)-6 and C-C motif chemokine ligand 2 (CCL2)/monocyte chemoattractant protein-1 (MCP-1), known to be upregulated following IgE-mediated monocyte stimulation,^16 20^ were also associated with more favorable RFS and OS (10 years) (figure 1C). These findings suggest that FcεRI-expressing immune cell infiltrates within HER2+ tumors, offer a chance for a therapeutic IgE to induce Fc-mediated effector functions and trigger pro-inflammatory mechanisms which may lead to improved outcomes for these patients.

**Figure 2:**
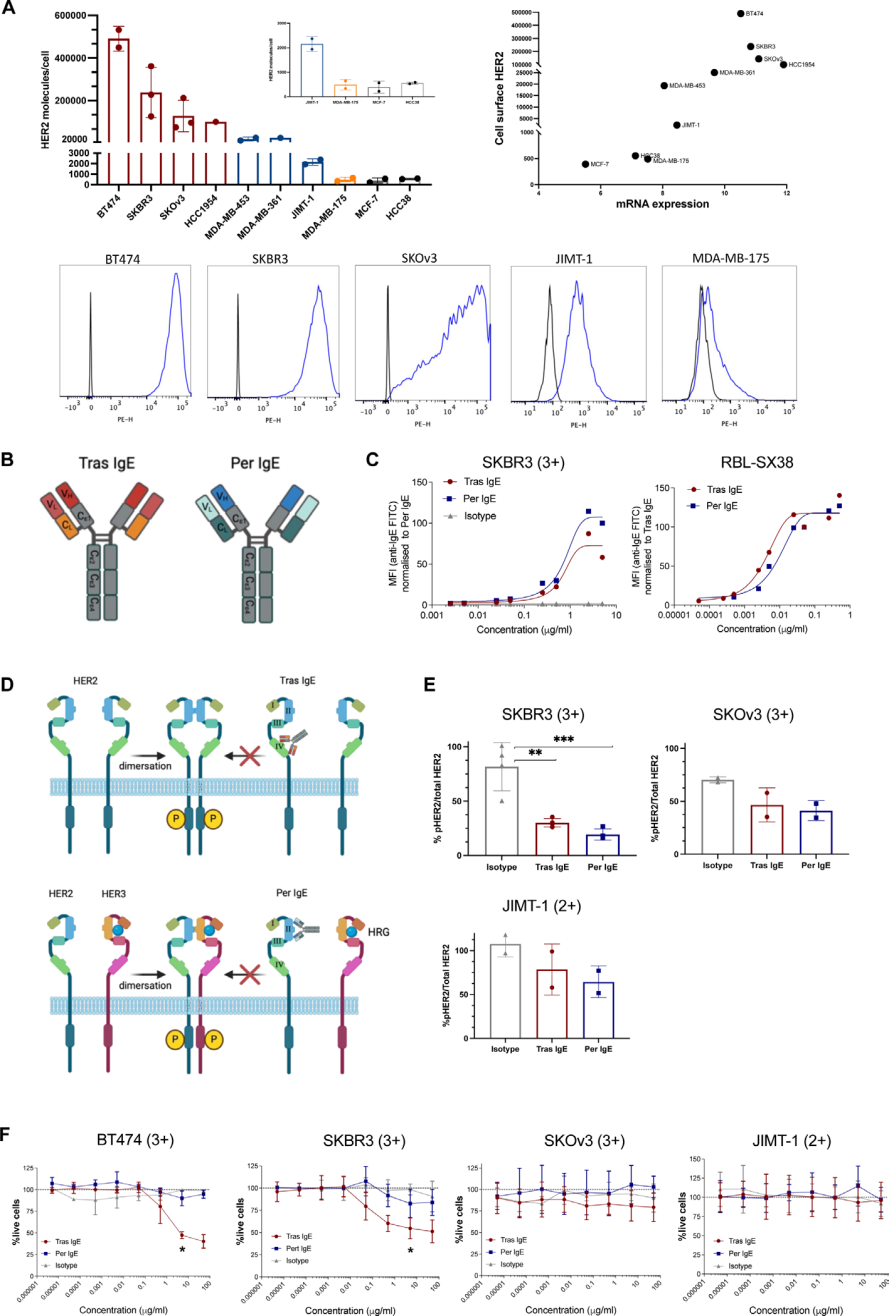
HER2 cell surface expression and retention of direct Fab-mediated effects by anti-HER2 IgE antibodies. (**A**) Left: HER2 expression levels of selected human cancer cell lines as determined by flow cytometry. Right: positive correlation of ERBB2 mRNA levels (DepMap) to flow cytometric determined HER2 cell surface levels (Spearman correlation, r=0.85, p=0.0029). Lower: representative flow cytometric histograms of HER2 cell-surface expression by different cancer cell lines as determined by anti-HER2 PE-staining (blue), in comparison to a non-specific isotype control (black). (**B**) Schematic of trastuzumab IgE (red) and pertuzumab IgE (blue). (**C**) Cell surface binding of Tras, Per and isotype control IgE antibodies to HER2 on SKBR3 cells (left) and to FcεRI-expressing RBL-SX38 cells (right). (**D**) Schematic of HER2 structure, dimerization and anti-HER2 monoclonal antibody Fab-mediated mechanisms of action. Trastuzumab binds to the extracellular domain IV of HER2 and inhibits ligand-independent homodimerization (HER2 with HER2). Pertuzumab recognizes the extracellular domain II of HER2 inhibiting ligand-dependent heterodimerization (HER2 with primarily HER3) in the presence of heregulin (HRG). (**E**) Impact of Tras IgE or Per IgE treatment on phosphorylation of HER2 in SKBR3 (n=3), SKOv3 (n=2) and JIMT-1 (n=2) cells. (**F**) Decreased ligand-independent cell proliferation of the two trastuzumab-sensitive HER2+ three cell lines, SKBR3 (n=3) and BT474 (n=3), on treatment with Tras IgE, but with not Per IgE. The antibodies had no effects on ligand-independent cell proliferation of the trastuzumab-resistant cell lines SKOv3 (HER2 3+) (n=4) and JIMT-1 (HER2 2+) (n=4). Mean of indicated independent experiments±SD (**A, E, F**) are shown. One-way analysis of variance (**E**), Kruskal-Wallis test (**F**). *p≤0.05; **p≤0.01; ***p≤0.001. Fab, fragment antigen-binding; HER2, human epidermal growth factor receptor 2; mRNA, messenger RNA; Per, pertuzumab; Tras, trastuzumab.

To select cellular models amenable to IgE treatment, we evaluated ten human breast and ovarian cancer cell lines for HER2 cell-surface expression by flow cytometry using a fluorescently-conjugated anti-HER2 antibody. The ranking based on levels of HER2 was consistent with the HercepTest 1+ to 3+ scores reported in the literature^24^,^26^ (referred to as HER2 1+ to 3+ throughout the manuscript). This included levels for HCC38 and MCF-7 which are reported to be HER2 0–1+. We found that these cells expressed low, yet still detectable levels of HER2 on the cell surface, similar to MDA-MB-175 which are classified as HER2 1+ (figure 2A left). We therefore classified MCF-7 and HCC38 as HER2 1+ for the purpose of this study. We furthermore confirmed the expression level classification by positive correlation (r=0.85) of reported HER2 messenger RNA expression (DepMap) with HER2 cell-surface expression levels detected by flow cytometry (figure 2A right).

The expected size of the produced Tras or Per IgE (figure 2B) was confirmed by SDS-PAGE (online supplemental figure 1A). Binding of both Tras and Per IgE to SKBR3 cancer cells (HER2 3+) and RBL-SX38 cells transgenically expressing human FcεRI, confirmed the ability to engage via the Fab and Fc regions, respectively (figure 2C).

Anti-HER2 IgG1 antibodies are known to inhibit HER2 dimerization, thereby impairing downstream phosphorylation in cancer cells.^2^ We determined whether these Fab-mediated activities were retained in the IgE versions (figure 2D). We observed a significant reduction of intracellular HER2 phosphorylation in SKBR3 cells in the presence of Tras or Per IgE, compared with cells without antibody, and to isotype control IgE-stimulated cells, with similar trends observed in SKOv3 and JIMT-1 cells (figure 2E). No significant differences in phosphorylation levels of extracellular signal-regulated kinase in the presence of Tras or Per IgE were observed in SKBR3 cells, potentially due to the ligand-independent nature of the assay (online supplemental figure 1B). Furthermore, Tras IgE, but not Per IgE, reduced ligand-independent cell proliferation of two HER2 3+ Tras-sensitive cell lines, BT-474 and SKBR3 (figure 2F). Neither Tras or Per IgE treatment showed any effects on the ligand-independent proliferation of SKOv3 (HER2 3+) or JIMT-1 (HER2 2+), consistent with a Tras-resistant phenotype (figure 2F). These findings suggest that Tras IgE can exert Fab-mediated effects against HER2+ Tras-sensitive cell lines, thus retaining the Fab-mediated effects of Tras and Per. However, SKOv3 and JIMT-1 cells, both carrying an activating mutation in PIK3CA (H1047R^27^ and C420R,^28^ respectively)*,* were resistant to ligand-independent anti-proliferative effects, as reported in the literature.^29^,^32^

Having demonstrated Fab-mediated direct effects of Tras and Per IgE, we next sought to determine their Fc-mediated immune effects in vitro. In co-cultures with nine HER2-expressing cancer cell lines, antigen-dependent cross-linking of Tras or Per IgE antibodies on RBL-SX38 cells yielded functional degranulation levels above those of non-specific IgE isotype controls.

**Figure 3:**
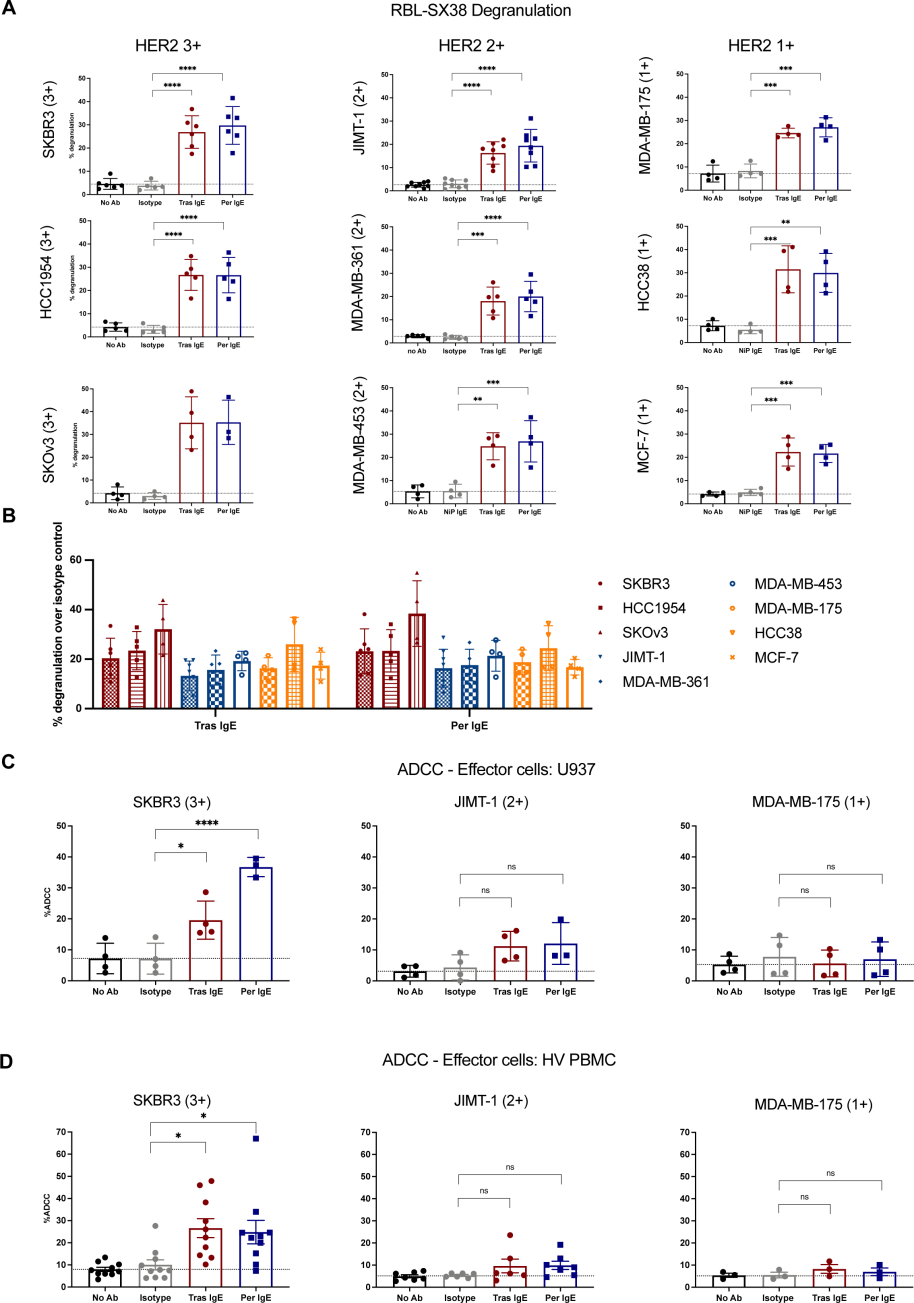
Fc-mediated effector functions by anti-HER2 IgE antibodies against cancer cell lines of various HER2 expression levels. (**A**) Antibody-induced degranulation of RBL-SX38 cells on target-specific cross-linking by HER2 3+ (SKBR3 (n=6), HCC-1954 (n=5), SKOv3 (n=4)), HER2 2+ (JIMT-1 (n=8), MDA-MB-361 (n=5), MDA-MB-453 (n=4)), HER2 1+ (MDA-MB-175 (n=4) cells, HCC38 (n=4) and MCF-7 (n=4)). (**B**) Degranulation levels were detected above isotype control for all assessed HER2-positive cell lines (HER2 3+ (red), HER2 2+ (blue), and HER2 1+ (orange). (**C–D**) Trastuzumab and pertuzumab IgE-mediated ADCC of SKBR3 (n=4), JIMT-1 (n=4) and MDA-MB-175 (n=4) cells by monocytic U937 cells (**C**), and of SKBR3 (n=10), JIMT-1 (n=5) and MDA-MB-175 (n=4) cells (**D**) by human peripheral blood mononuclear cells. Mean of indicated independent experiments±SD (**A**–**C**) or SEM (**D**) are shown. One-way analysis of variance (**A**, **B**, **C**, **D** (except SKBR3)), Kruskal-Wallis test (**B**, **D** (SKBR3)). *p≤0.05; **p≤0.01; ***p≤0.001; ****p≤0.0001; ns non-significant. ADCC, antibody-dependent cell-mediated cytotoxicity; HER2, human epidermal growth factor receptor 2.

The levels of degranulation were comparable between all 1+ to 3+cell lines, and thus irrespective of HER2 expression levels (figure 3A,B). No degranulation was triggered by WM1366 melanoma cancer cells devoid of HER2 expression (online supplemental figure 2A). We selected one cell line representative of each HER2 expression level, namely SKBR3 (HER2 3+), JIMT-1 (HER2 2+) and MDA-MB-175 (HER2 1+), for tumor cell killing assays. In these, we measured the levels of tumor cell ADCC by human U937 monocytic cells (figure 3C) and by primary human PBMCs (figure 3D) mediated by Tras and Per IgE. For both IgE antibodies, the level of ADCC triggered above isotype control IgE, was highest against SKBR3 (HER2 3+) cells. While not statistically significant, some cytotoxicity was observed against JIMT-1 (HER2 2+) cells, whereas no cytotoxicity of MDA-MB-175 (HER2 1+) cells was triggered. No significant phagocytosis was observed for any of the target cells studied (online supplemental figure 2B, C). These data demonstrate Fc-mediated functions of Tras and Per IgE against HER2-expressing cancer cells.

### Tras and Per IgE trigger an Fc-mediated pro-inflammatory shift in human monocytes ex vivo

**Figure 4:**
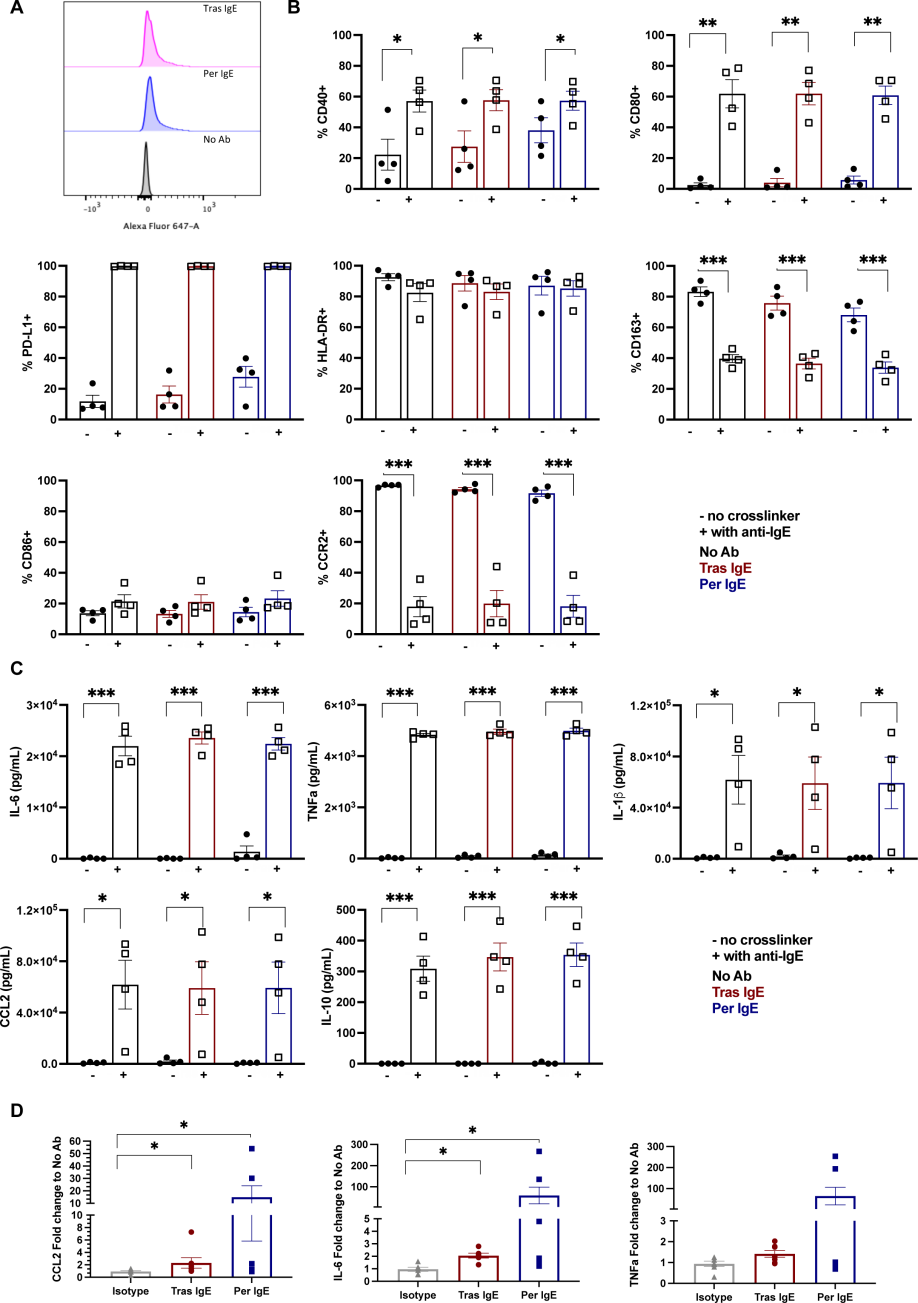
IgE Fc-mediated a pro-inflammatory shift in human monocyte phenotype and secreted mediators ex vivo. (**A**) Binding of Alexa Fluor 647-labeled Tras and Per IgE to primary monocytes confirmed available FcεRs to bind IgE. (**B**) Cross-linking of Tras and Per IgE with anti-IgE on the monocyte cell surface triggers changes in expression levels of cell surface markers. Markers evaluated were CD40, CD80, PD-L1, HLA-DR, CD163, CD86 and CCR2 (n=4). (**C**) Changes in pro-inflammatory and anti-inflammatory cytokine secretion by primary monocytes on cross-linking of Tras and Per IgE with polyclonal anti-IgE (n=4). (**D**) Changes in cytokine secretion in co-cultures of human peripheral blood mononuclear cells and SKBR3 (HER2 3+) cells after 3 hours in the presence of anti-HER2 IgE antibodies, compared with isotype control. Mean of indicated independent experiments±SEM are shown. Multiple t-test (**B, C**), Kruskal-Wallis test (**D**). *p≤0.05; **p≤0.01; ***p≤0.001; ****p≤0.0001; ns non-significant. CCL2, C-C motif chemokine ligand 2; HER2, human epidermal growth factor receptor 2; HLA-DR, human leukocyte antigen-DR; IL, interleukin; PD-L1, programmed death-ligand 1; Per, pertuzumab; Tras, trastuzumab.

We previously reported that IgE antibodies specific for melanoma and ovarian cancer antigens stimulate pro-inflammatory activation of immune effector cells,^15 16^ however, this has not yet been evaluated with anti-HER2 IgEs or in the breast cancer context. We first confirmed the binding of Tras and Per IgE to primary monocytes (figure 4A). In ex vivo stimulation assays, cross-linking of Tras and Per IgE with a polyclonal antibody to mimic immune complex formation on the surface of human primary monocytes, resulted in significant upregulation of cell-surface pro-inflammatory, antigen presentation and T-cell activation markers CD40, CD80, and the programmed death-ligand 1 (PD-L1) checkpoint protein, while levels of human leukocyte antigen (HLA)-DR expression remained unchanged. Simultaneously, monocyte stimulation by Tras and Per IgE resulted in significant downregulation of the alternatively activated monocyte (M2-like) marker CD163 and a modest upregulation of the M1 marker CD86. Furthermore, the monocyte chemoattractant receptor CCR2 was downregulated, consistent with stimulation by the monocyte chemoattractant CCL2/MCP1 (figure 4B).^15 16 20^ Supernatants of these co-cultures showed significantly increased levels of pro-inflammatory cytokines IL-6, TNF-α, IL-1β. Consistent with previous reports on other tumor-targeting IgE antibodies,^15 16 20^ anti-HER2 IgE cross-linking, also stimulated upregulation of CCL2, and of the anti-inflammatory mediator IL-10 (figure 4C).

Next, we assessed pro-inflammatory cytokine release by human PBMCs in the presence of HER2-expressing tumor cells and Tras or Per IgE. In concordance with our findings above, we measured increased levels of CCL2/MCP1, IL-6 and TNF-α following antigen-specific cross-linking of Tras and Per IgE on primary immune cells by co-culture with SKBR3 cells (figure 4D).

This data taken together demonstrates cross-linking of Tras and Per IgE on human monocytes significantly induced upregulation of immune-stimulatory cell-surface markers and downregulation of scavenger receptors, alongside the production of pro-inflammatory immune mediators.

### Tras and Per IgE do not potentiate basophil activation in unfractionated human blood

**Figure 5:**
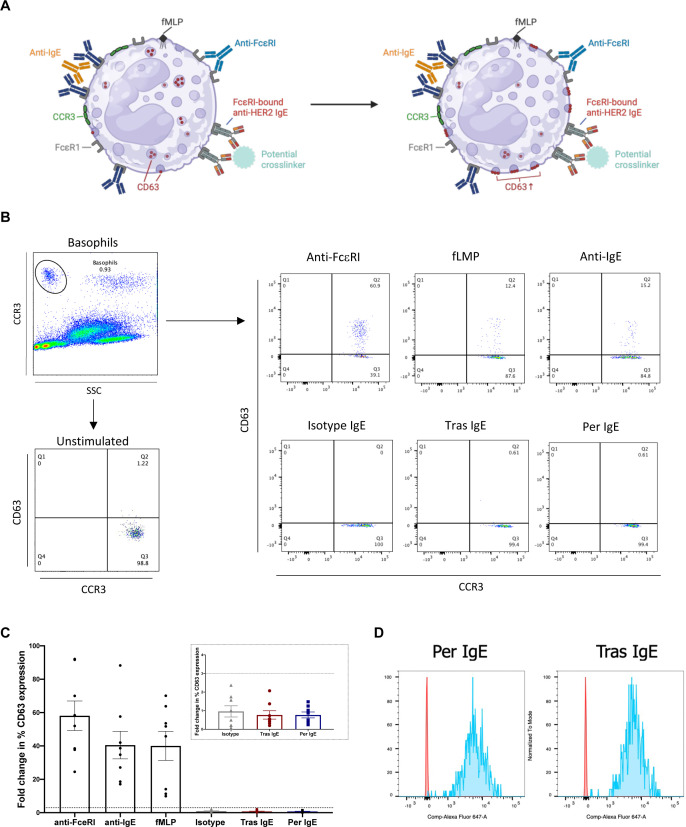
Anti-HER2 IgE antibodies do not activate basophils ex vivo. (**A**) Schematic of the basophil activation test. Immune stimulation ex vivo triggers basophil activation as measured by cell surface CD63 upregulation. (**B**) Gating strategy and representative flow cytometry plots for positive control immune stimuli, and Tras and Per IgE antibodies. (**C**) Basophil activation was triggered on ex vivo stimulation of human whole blood samples with positive immune stimuli, but not by isotype IgE control, Tras or Per IgE antibodies (n=8). Mean of indicated independent experiments±SEM are shown. (**D**) Binding of Alexa Fluor 647-labeled Tras and Per IgE to whole blood basophils to confirm available FcεRs on the surface of these cells were available to bind exogenous IgE. HER2, human epidermal growth factor receptor 2; Per, pertuzumab; Tras, trastuzumab.

A key concern with the clinical application of IgE immunotherapy is the potential of IgE bound to blood basophils or mast cells to encounter agents in the circulation able to cross-link IgE and stimulate cell degranulation, resulting in type 1 hypersensitivity and systemic anaphylaxis. We employed the BAT, normally used in allergy to predict and monitor such reactions to drugs, including therapeutic antibodies,^1733^,^35^ to assess the potential of Tras or Per IgE to trigger type 1 hypersensitivity (figure 5A). Multivalent ligand stimulation by immune control stimuli (IgE-mediated anti-FcεRI and anti-IgE, and non-IgE mediated, fMLP) activated CCR3^high^SSC^low^ basophils in whole unfractionated blood samples from eight healthy volunteers, as measured by upregulation of CD63 cell-surface expression. However, no activation was triggered by either Tras or Per IgE (figure 5B,C). We confirmed that circulating basophils had free unoccupied Fcε-receptors with capacity to bind Tras or Per IgE, as demonstrated by binding of Alexa Fluor 647-conjugated IgE antibodies (figure 5D).

Absence of basophil activation in whole human blood stimulated with Tras and Per IgEs provides an early indication for absence of type 1 hypersensitivity to either of these antibodies.

### Tras IgE restricts growth of high HER2+ (HER2 3+) and medium HER2+ (HER2 2+) trastuzumab-resistant tumors

An unmet need in HER2+ cancers is an effective therapy for tumors with resistance to Tras Fab-mediated effects. Since resistant tumors often retain HER2 expression, we hypothesized that Tras IgE may harness key effector functions against HER2+ tumors in the absence of Fab-mediated signaling effects by the antibody. In vitro evaluations pointed to Tras IgE functionality against high/medium HER2+ cells (figure 3). We therefore evaluated the effects of Tras IgE in high and medium HER2+ (HER2 3+/2+) Tras-resistant cancers in vivo (model development shown in online supplemental figure 3).

**Figure 6:**
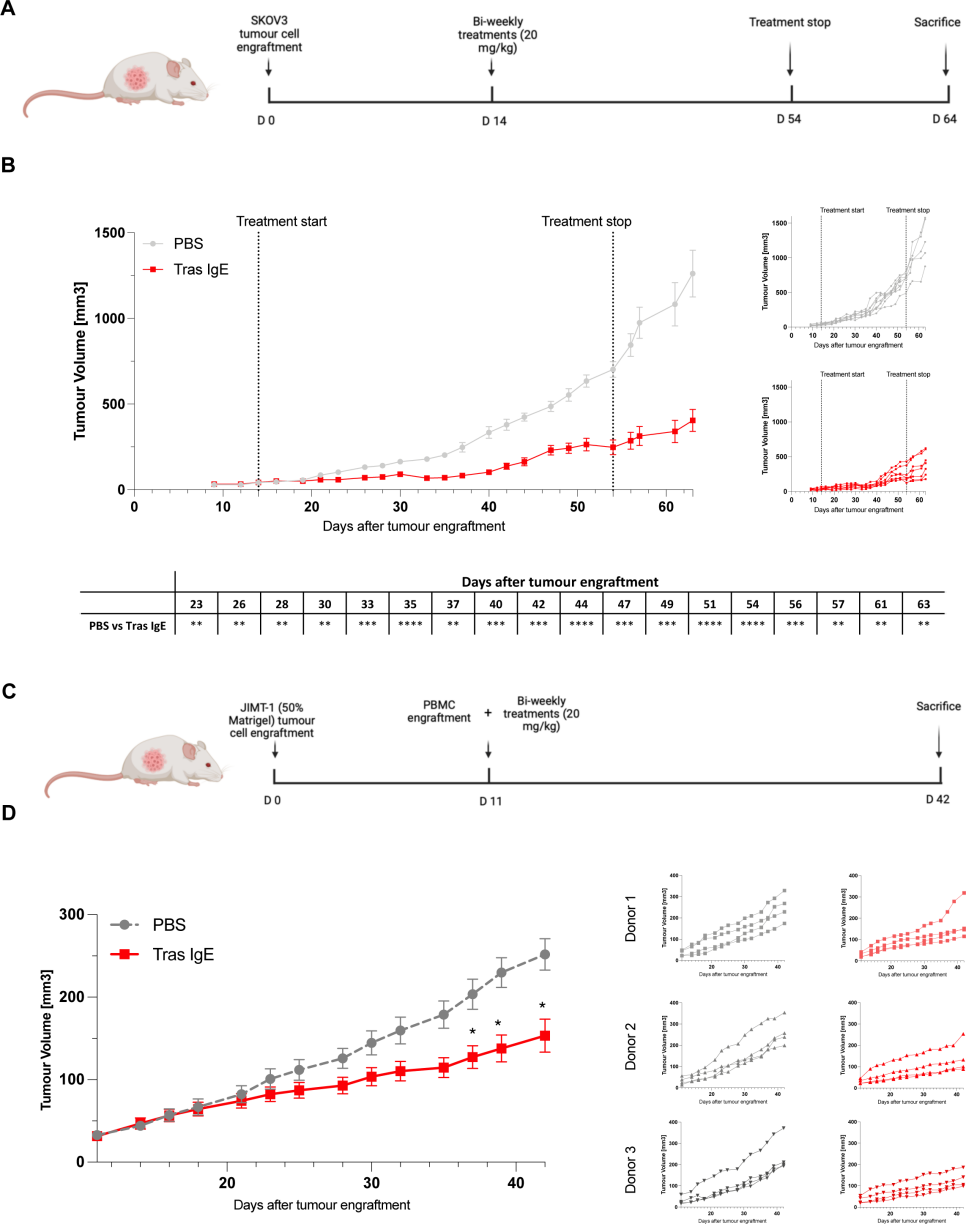
In vivo efficacy of Tras IgE against trastuzumab-resistant high and medium HER2-expressing tumors. (**A**) Design of in vivo Tras IgE efficacy study against high HER2 (3+), trastuzumab-resistant tumors. (**B**) Left: SKOv3 tumor growth restriction was observed in Tras IgE-treated mice, compared with untreated (PBS) (n=8 per group). Vertical dotted lines indicated start and end of IgE treatment (day 14 and 54, respectively). Right: tumor growth curves for individual mice. Bottom: statistically significant differences in tumor volumes between Tras IgE and PBS control-treated mice. (**C**) Design of in vivo Tras IgE efficacy study against medium HER2 (2+), trastuzumab-resistant tumors. (**D**) Left: JIMT-1 tumor growth restriction was observed in Tras IgE-treated mice (n=12) compared with controls (PBS, n=12) (combined data for mice engrafted with human PBMC donors 1–3). Right: tumor growth curves for individual mice engrafted with human PBMCs from donors 1, 2 and 3. Mean±SEM are shown. Mixed-effects analysis (**B**) and Multiple t-test (**D**). *p≤0.05; **p≤0.01; ***p≤0.001; ****p≤0.0001; ns non-significant. HER2, human epidermal growth factor receptor 2; PBMC, peripheral blood mononuclear cell; PBS, phosphate-buffered saline; Per, pertuzumab; Tras, trastuzumab.

SKOv3 cells were used to establish high HER2+ (HER2 3+), Tras-resistant tumors in immunodeficient mice (NOD-Prkdc^em26Cd52^ll2rg^emCd22^) engrafted with human hematopoietic CD34^+^ stem cells (hu-mice). Tras IgE showed no Fab-mediated effects against high HER2+ (HER2 3+) SKOv3 cells (figure 2F), however, it could engender SKOv3 cytotoxic killing by HV PBMCs (online supplemental figure 4A). Bi-weekly intravenous administration with Tras IgE (20 mg/kg) was evaluated in hu-mice (figure 6A,B, online supplemental figure 4B). Tras IgE significantly restricted tumor growth, which was sustained for more than a week after completion of dosing (tumor growth inhibition (TGI)=71.5%, figure 6B). In the PBS control group, three out of eight were euthanized before or on treatment completion (D54), primarily due to tumor condition. Only one mouse treated with Tras IgE was euthanized before study completion (D63), suggesting a less aggressive disease phenotype.

We next evaluated the potential of Tras IgE to target medium HER2+ Tras-resistant tumors in mice engrafted with human PBMCs (online supplemental figure 4C). Human JIMT-1 (HER2 2+) cells are known to be resistant to Tras,^28^ and we confirmed that neither Tras IgE nor Per IgE impacted JIMT-1 cell proliferation in vitro (figure 2F). JIMT-1 tumor-bearing mice were engrafted with human PBMCs from healthy donors and treated with 20 mg/kg of Tras IgE bi-weekly (figure 6C). Despite only triggering low levels of tumor cell killing in vitro, Tras IgE treatment led to a significant reduction in JIMT-1 (HER2 2+) tumor volume (TGI=36.6%), as compared with PBS controls (figure 6D).

These results demonstrate significant tumor growth restriction of Tras-resistant HER2+ tumors with Tras IgE treatment, indicating Fc-mediated immune functions in the absence of direct Fab-mediated effects in vivo.

### Evidence of TME remodeling following Tras IgE treatment

The TME greatly impacts response to chemotherapy, targeted and immune therapies for cancer,^10 33^ thus we investigated the impact of Tras IgE treatment on the TME in high and medium HER2+ Tras-resistant tumors.

**Figure 7:**
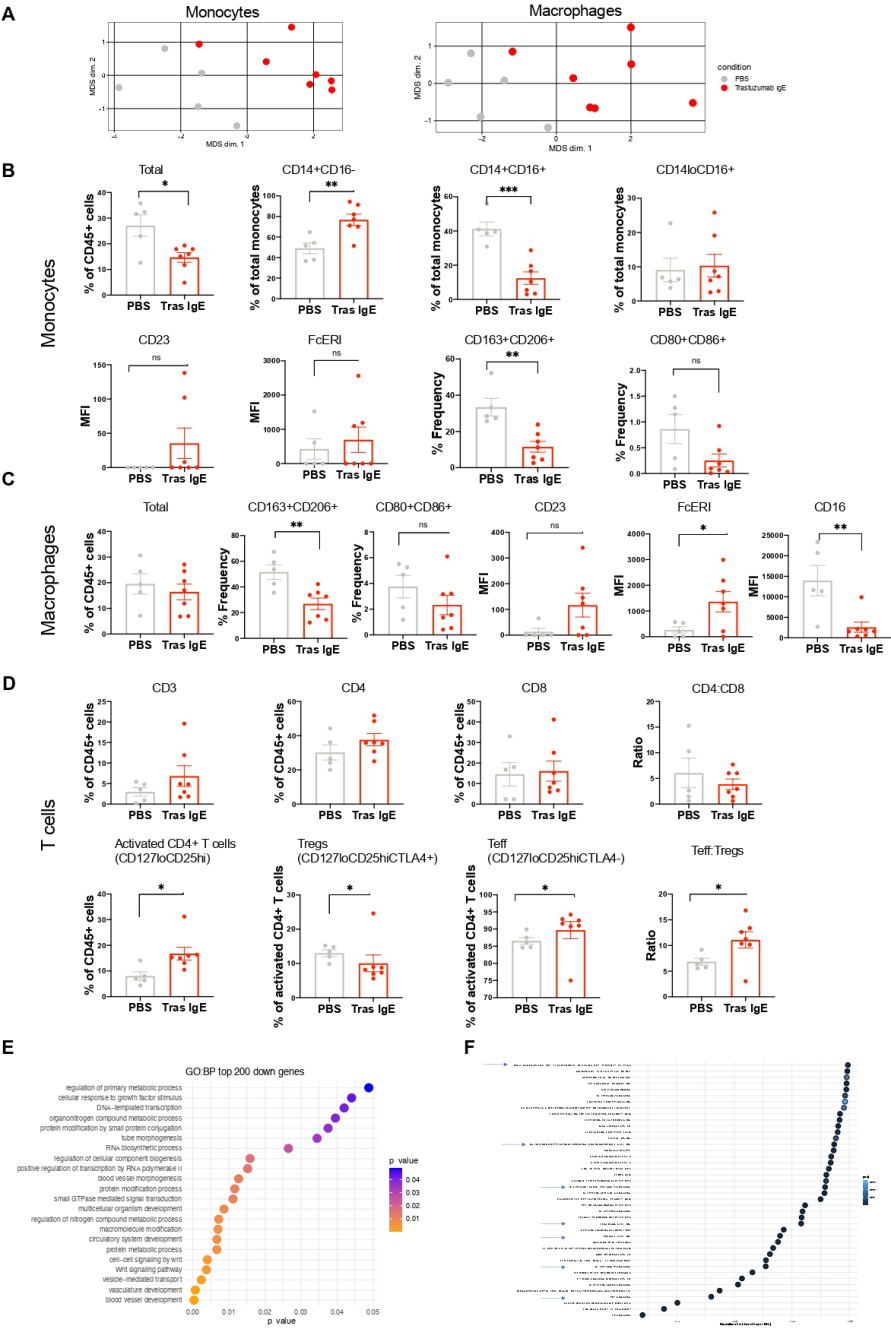
Immunostimulatory and anti-tumoral effects of Tras IgE in the TME. (**A–D**) Intratumoral immune cell populations in TME of high human epidermal growth factor receptor 2, trastuzumab-resistant tumors of treated (Tras IgE, n=7) and untreated (PBS, n=5) mice, as determined by flow cytometry. (**A**) Left: MDS plot for intratumoral monocyte signatures, Right: MDS plot for intratumoral macrophages. Each dot represents an individual tumor. (**B**) Flow cytometric evaluations of intratumoral monocytes and percentages of the classical (CD14^+^CD16^−^), intermediate (CD14^+^CD16^+^) and non-classical monocyte (CD14^lo^CD16^+^) subsets (top panel). Fcε receptor expression on intratumoral monocytes, CD163^+^CD206^+^ and CD80^+^CD86^+^ monocyte infiltrating populations (bottom panel) from treated (Tras IgE) and untreated (PBS) tumors. (**C**) Levels of total intratumoral macrophages, alternatively-activated macrophages M2 (CD163^+^CD206^+^), M1-like (CD80^+^CD86^+^), as well as FcεR and CD16 expression on intratumoral macrophages in treated (Tras IgE) and untreated (PBS) tumors determined by flow cytometry. (**D**) Flow cytometric evaluations of levels of intratumoral total T cells (CD3^+^), T helper cells (CD4^+^) and cytotoxic T cells (CD8^+^), and CD4:CD8 ratio, CD4^+^ T cells, Tregs and Teff cells, and Teff:Treg ratio, in treated (Tras IgE) and untreated (PBS) tumors. (**E**) Gene overexpression analysis of top 200 downregulated genes in tumors from Tras IgE treated (n=6) compared with untreated (PBS, n=6) mice. (**F**) Downregulation of pathways in Tras IgE treated (n=6) compared with untreated (n=6) tumor samples were determined by Gene Set Enrichment Analysis (Reactome). Only pathways with p value adjusted ≤0.05 and normalized enrichment score ≤−2 are shown. Mean±SEM is shown (**B–D**). Unpaired t-test (**B–D**). *p≤0.05; **p≤0.01; ***p≤0.001; ns non-significant. MDS, multidimensional scaling; MFI, mean fluorescence intensity; NES, normalized enrichment scores; PBS, phosphate-buffered saline; Per, pertuzumab; Teff, effector T cells; TME, tumor microenvironment; Tras, trastuzumab; Treg, regulatory T cells.

We studied immune cell populations within tumor-infiltrating hCD45^+^ cells from SKOv3 xenografts excised at the end of study. We investigated phenotypic changes in different intratumoral myeloid subsets within the TME (figure 7A–C, gating strategy online supplemental figure 5A). Monocyte and macrophage population characteristics were distinct in Tras IgE-treated tumors compared with untreated (PBS) controls (figure 7A). Despite an overall reduction in monocytes in tumors from treated compared with untreated mice, we found significant enrichment of classical monocyte (CD14^+^CD16^−^) and reduced intermediate monocyte (CD14^+^CD16^+^) populations with Tras IgE treatment, while non-classical monocyte (CD14^lo^CD16^+^) populations were comparable. FcεR expression on monocytes remained unchanged with IgE treatment. No differences in CD80^+^CD86^+^ monocyte populations were observed, however, CD163^+^CD206^+^ alternatively-activated monocyte populations were reduced in tumors from the Tras IgE group compared with controls (figure 7B).

Similarly, while the proportion of total tumor-infiltrating macrophages was comparable between treatment groups, Tras IgE-treated mice showed lower levels of alternatively activated CD163^+^CD206^+^, but comparable levels of classically-activated CD80^+^CD86^+^ macrophages, supporting a shift away from immunosuppression. Macrophages in the Tras IgE treatment group showed upregulated expression of FcεRI, similar levels of CD23 expression, and downregulated expression of CD16 (figure 7C). Tras IgE treatment had no significant impact on the proportion of tumor-infiltrating CD3^+^, CD4^+^ or CD8^+^ T cells, or on CD4:CD8 ratios (figure 7D, gating strategy online supplemental figure 5B). However, higher densities of activated CD4^+^ (CD127^lo^CD25^hi^) T cells were found in tumors from IgE-treated mice compared with controls. Within this population, higher effector T cell (Teff) to regulatory T cell (Treg) ratios (Teff cells: CD127^lo^CD25^hi^CTLA4^−^ and Treg-like cells: CD127^lo^CD25^hi^CTLA4^+^) were measured in tumors from mice treated with Tras IgE.

Overall, Tras IgE was associated with remodeling of tumor-infiltrating monocyte, macrophage and T-cell populations towards more immunostimulatory phenotypes within the TME of HER2-expressing, Tras-resistant tumors.

Tumors excised at the end of study of the medium HER2 (HER2 2+) Tras-resistant cancer model exhibited limited immune cell infiltration despite human PBMC engraftment measured in the spleen (online supplemental figure 4C, D). To evaluate the effects of Tras IgE treatment on the TME in this model, we conducted transcriptomic analyses of excised tumors. Gene over-representation analysis showed significant downregulation of several cancer-promoting biological processes, such as reduction in pathways associated with Wnt-signaling and blood vessel development, in the tumors from Tras IgE treated mice, as compared with controls (figure 7E). Furthermore, GSEA performed using Reactome (filtered by p value adjusted ≤0.05 and then by normalized enrichment score ≤−2) revealed that Tras IgE treatment was associated with downregulation of anti-inflammatory or immunosuppressive pathways, such as Treg regulation, IL-4 and IL-13 signaling, IL-10 signaling, and programmed cell death protein 1 (PD-1) signaling (figure 7E; complete GSEA results in online supplemental figure 6). Furthermore, in concordance with known immune-stimulating roles of IgE in anti-parasitic responses,^36^ and previous observations of tumor-targeting IgEs affecting such mechanisms,^16^ we observed downregulation of several pathways related to parasite infections in tumors treated with Tras IgE. These findings support Tras IgE-mediated activation of immune activation pathways against HER2-expressing breast cancer.

Taken together, changes from immunosuppressive to immunostimulatory processes were observed in the TME of two distinct HER2+Tras-resistant tumors following in vivo treatment with Tras IgE. These data indicate that the efficacy of Tras IgE may exert immune cell-mediated killing and pro-inflammatory immune-stimulating effects.

## Discussion

HER2 overexpression in cancer is linked to rapid progression and poor prognosis.^1 37 38^ Despite availability of several HER2-targeted therapies, including mAbs, antibody-drug conjugates (ADCs) and tyrosine kinase inhibitors (eg, lapatinib), lack of therapeutic response in patients due to intrinsic or acquired resistance is commonly observed.^3^,^5^ In many cases of intrinsic or acquired Tras resistance, tumors retain some cell-surface HER2 expression.^4^ This might allow for an HER2-targeted approach that does not rely on blockade of the HER2 downstream signaling network. Furthermore, an immunosuppressive TME might limit the ability of current IgG-based immunotherapeutic agents to elicit their full anti-tumoral potential. These challenges may require alternative antibodies which are able to: (1) operate in Th2-biased immune conditions such as those of tissue-residing tumors, and (2) activate a distinct arm of the immune system not accessed by currently available treatments to promote pro-inflammatory conditions in the TME.

We previously reported the Fc-mediated functions of anti-tumor IgEs,^15 16 18 39^ including of Tras IgE.^18^ In the present study, we studied, for the first time, effector cell pro-inflammatory mechanisms triggered by anti-HER2 IgEs and investigated how these effects are directed against HER2-expressing cancer cells, including those resistant to Tras Fab-mediated effects. We generated Tras and Per with human IgE Fc regions as proof-of-concept. Both antibodies retained the ability to bind cell-surface HER2 and to reduce levels of HER2 phosphorylation in the presence of a ligand, a major mechanism of efficacy of these antibodies. As expected, this was also the case for Tras-resistant HER2 cells, as resistance is often conferred through mutations further downstream.^3^,^528^ While Tras IgE showed ligand-independent inhibition of cell proliferation in Tras-sensitive cell lines, Per IgE, known to inhibit ligand-dependent heterodimerisation,^2^ did not. This confirmed the retention of dimerization-inhibition specificity of these antibodies when generated in an IgE format.

We further demonstrated induction of degranulation of a basophilic cell line by the anti-HER2 IgEs, on cross-linking with HER2-positive cancer cells (HER2 +1 to +3). These findings are largely consistent with previously reported degranulation with the melanoma targeting anti-CSPG4 IgE antibody.^15^ In contrast, Tras and Per IgE mediated ADCC against HER2-expressing cancer cells in an antigen-density dependent manner, an observation in accordance with previous reports with the anti-FRα MOv18 IgE antibody targeting ovarian cancer.^16^ These differences in the reliance on antigen density for degranulation and ADCC effects are yet to be understood and may, for example, stem from differences in FcεR density.^11^ Further research is required to elucidate the minimum requirement for IgE-Fc mediated effector functions and the potential to harness these against medium to low antigen-expressing cancers.

While Fc-mediated killing of medium-expressing HER2+ cancers is important for the anti-tumoral effect of tumor antigen-specific IgE in vivo, the mechanism of action of anti-HER2 IgE in vivo may include both Fc-mediated ADCC as well as priming of immune cells towards a pro-inflammatory state as we previously reported.^15 20^ Here we confirmed that cross-linking of anti-HER2 IgE antibodies on primary human monocytes resulted in phenotypic changes, including upregulation of CD40, CD80 and CD86 and downregulation of CD163. These changes were accompanied by enhanced secretion of the pro-inflammatory mediators TNF-α, IL-1β, IL-6 and of the monocyte chemoattractant MCP-1. Similar trends were seen on antigen-specific cross-linking after only 3 hours. These findings, consistent with previous reports with IgE antibodies targeting other tumor-associated antigens such as CSPG4, SLC3A2 and FRα,^15 16 20 39^ confirmed the ability of anti-HER2 IgEs to engender a pro-inflammatory shift of monocytes, and point to a potential mechanism for Fc-meditated stimulation of antitumor immune conditions.

Having demonstrated anti-tumoral functionality of the two anti-HER2 IgEs in vitro/ex vivo, we aimed to address the common concern of safety when using IgEs as therapeutics, due to the role of IgE in allergic responses. We employed the BAT conducted in whole unfractionated blood, allowing for the presence of any potential modulators in circulation able to cross-link and activate basophils to degranulate and thus to potentiate type I hypersensitivity.^33^,^3540^ As previously reported for other tumor antigen-specific IgEs,^14 15 40 41^ no activation of basophils was observed by either antibody, despite clear binding of the antibodies to FcεRs on the cell surface. Results of this assay have also been used as exclusion criteria in the phase I clinical trial of MOv18 IgE in patients with FRα-expressing tumors. Patients with negative BAT reactivity results to MOv18 IgE showed no signs of anaphylaxis, whereas the assay predicted the single case of anaphylaxis in the trial.^17^ Therefore, the lack of basophil activation by the two tested anti-HER2 antibodies may be interpreted as an early indication of lack of type I hypersensitivity reaction with anti-HER2 administration in humans.

Building on the immune-mediated and immunomodulatory functions of IgE we observed in vitro and ex vivo, we demonstrated tumor growth inhibition by Tras IgE in two Tras-resistant models; one with high HER2 (HER2 3+) and one with medium HER2 (HER2 2+) expression levels. We further hypothesized that anti-HER2 IgE treatment may trigger pro-inflammatory activation and repolarization of otherwise immunosuppressive effector cells in the TME, through Fc-mediated mechanisms. In both models, treatment with Tras IgE in vivo resulted in significant changes in the TME. In high HER2 (HER2 3+) Tras-resistant tumors, total macrophage or T-cell infiltration remained mostly unchanged following systemic treatment with IgE. Consistent with in vitro findings, tumors from treated animals showed significantly higher levels of classical monocytes, and lower levels of intermediate monocytes. Intermediate monocytes have previously been shown to be more prevalent in peripheral blood samples of patients with early-stage breast cancer compared with healthy individuals and are thought to contribute to tumorigenesis.^42^ Similarly, peripheral blood monocyte populations in patients with chemotherapy-naive ovarian cancer showed significant increase in intermediate monocytes at the cost of classical monocytes. The non-classical monocyte population remained unchanged. This increase in intermediate blood monocytes was positively associated with tumor burden in the peritoneum, thus progression, and correlated with a protumoral and immunosuppressive microenvironment in ascites. Furthermore, there was a clear link between intermediate monocyte expansion, reduction in Teff:Treg ratio and natural killer cells, and an increase in CCR2^+^ and CD163^+^CD206^+^ macrophages.^43^ These findings are consistent with observations in our study of HER2 3+ tumors, showing infiltration of CD163^+^CD206^+^ macrophages in untreated tumors, and a significant reduction of this immunosuppressive subset through treatment with Tras IgE. This is in accordance with recent data demonstrating M2-like macrophage and tumor-associated macrophage (TAM) repolarization to pro-inflammatory phenotypes by tumor-antigen specific IgE.^16 39 41^ Here, we further demonstrated that Tras IgE treatment induced a shift in CD4^+^-activated T cells from Treg toward Teff cells in the TME in vivo, resulting in an increase in Teff:Treg ratio. This observation may signify the ability of IgE immunotherapy to support a pro-inflammatory immune microenvironment, a potential effect that reaches beyond the immediate Fc-mediated effects on TAMs.

In contrast to the high HER2+ tumor model, immune infiltration was limited in the medium HER2+ Tras-resistant model, pointing to a more immune “cold” tumor. These tumors are notoriously hard to treat, as the potential of therapeutics to elicit immune responses in the TME may be limited. However, at the transcriptomic level, we observed a significant downregulation of signaling pathways involved in Treg development, checkpoint PD-1 signaling, and IL-10 signaling in Tras IgE-treated animals compared with untreated mice. These findings indicate a reduction in immunosuppressive mechanisms in the TME following Tras IgE treatment. Interestingly, excised tumors from anti-HER2 IgE-treated mice exhibited downregulation of IL-4 and IL-13 signaling pathways, which are markers of a Th2 response and are often associated with allergic responses and IgE. This additionally highlights a shift away from immunosuppressive towards an immune active and less immunocompromised TME. It furthermore refutes the notion that the use of anti-tumoral IgE will enhance Th2 responses, thereby leading to allergic-like immunity. Instead, Tras IgE treatment was associated with activation of immune pathways associated with anti-parasite responses, suggesting that in the cancer therapy context, IgE can activate an arm of the immune responses normally harnessed to clear pathogens. These findings are consistent with our previous studies in which we demonstrated a shift towards an immunoactive TME with IgE immunotherapy in melanoma and ovarian cancer contexts.^15 16^ Aside from immunomodulatory effects, Tras IgE treatment in these medium HER2-expressing, Tras-resistant tumors also led to a downregulation of pathways directly associated with tumorigenesis, such as blood vessel development^44^ and Wnt signalling,^45^ pointing to the impact of IgE-mediated immunomodulation against cancer-promoting mechanisms.

Future studies may confirm retention of anti-HER2 IgE binding to cells from excised tumors at the end of study. An in-depth investigation of the effects of anti-HER2 IgEs on several key signaling pathways such as PI3K/Akt, both in vitro and in excised tumors from these in vivo models, would also be valuable. This may give further insights into the overall mechanisms of action of these anti-HER2 IgEs and confirm the lack of Fab-mediated effects in excised tumors from these in vivo models. Finally, there may also be a potential to administer anti-HER2 IgE in combination with anti-HER2 IgG1. This therapeutic approach at an early stage of disease may lead to an immunological response with protective effects.

Taken together, we show that anti-HER2 IgEs engender significant Fc-mediated anti-tumoral effects in vitro, ex vivo and in vivo against HER2-expressing Tras-resistant cancer. We demonstrate beneficial immune cell-mediated and immunomodulatory effects of anti-HER2 IgE, especially promotion of pro-inflammatory immune phenotype shifts and “reprogramming” of the TME in HER2+ cancers, reducing immunosuppressive effects and protumoral pathways. These highlight the potential for use of anti-HER2 IgE antibodies to stimulate pro-inflammatory and antitumor mechanisms against HER2-expressing tumors with resistance to current HER2 targeting therapies.

## Supplementary material

10.1136/jitc-2024-010945 online supplemental file 1

## Data Availability

All data relevant to the study are included in the article or uploaded as supplementary information.
