# Supplementary material for: Fc-mediated immune stimulating, pro-inflammatory and antitumor effects of anti-HER2 IgE against HER2-expressing and trastuzumab-resistant tumors
Source: J Immunother Cancer. 2025 Mar 12;13(3):e010945. doi: 10.1136/jitc-2024-010945 (PMC12010294; doi:10.1136/jitc-2024-010945)
Supplement: online supplemental file 1 [file jitc-13-3-s001.pdf]

SUPPLEMENTARY FIGURES

Suppl. Figure 1

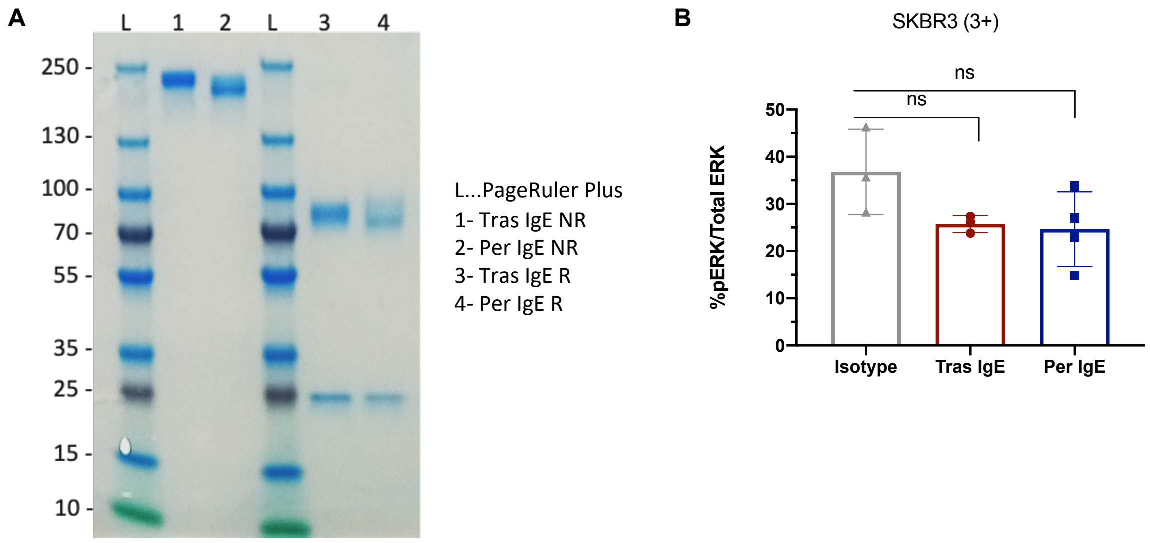

Supplementary Figure 1: Antibody quality and impact of anti-HER2 on ERK phosphorylation levels. A) SDS-PAGE of fully assembled IgE antibodies (around 190 kDa) in non-reduced (lanes 1 & 2; NR) and heavy and light chain (approx. 75 and 25 kDa, respectively) under reduced (lanes 3 & 4; R) conditions. B) Phosphorylation levels of ERK, measured using an HTRF phospho-ERK assay, upon treatment with Tras IgE or Per IgE. Mean of indicated independent experiments ± SD (B) is shown. One way ANOVA (B); ns non-significant.

Suppl. Figure 2

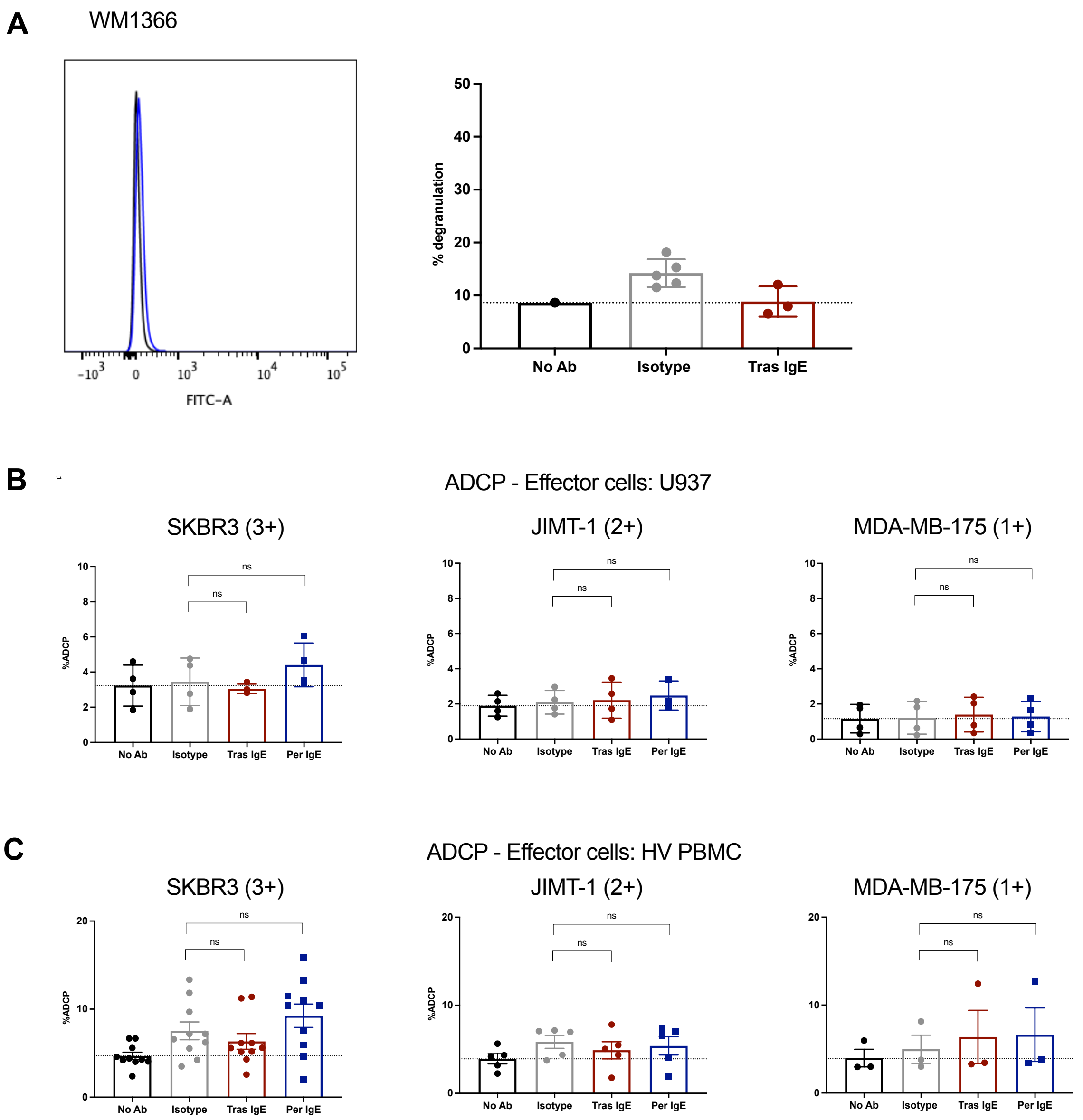

Supplementary Figure 2:_Fc-mediated effector functions by anti-HER2 IgE antibodies against cancer cell lines of various HER2 expression levels. A) Left: Histogram of binding of anti-HER2 (blue) and isotype control (black) antibodies to the melanoma cell line WM1366. Right: lack of antibody-induced degranulation of RBL-SX38 cells upon target-specific crosslinking to WM1366. B) Tras and Per IgE-mediated ADCP of SKBR3 (n=4), JIMT-1 (n=4) and MDA-MB-175 (n=4) cells by monocytic U937 cells, and C) of SKBR3 (n=10), JIMT-1 (n=5) and MDA-MB-175 (n=3) cells by healthy volunteer PBMCs. Mean of indicated independent experiments ± SD (A-B) or SEM (C) is shown. One-Way ANOVA (B, C); ns non-significant.

Suppl. Figure 3

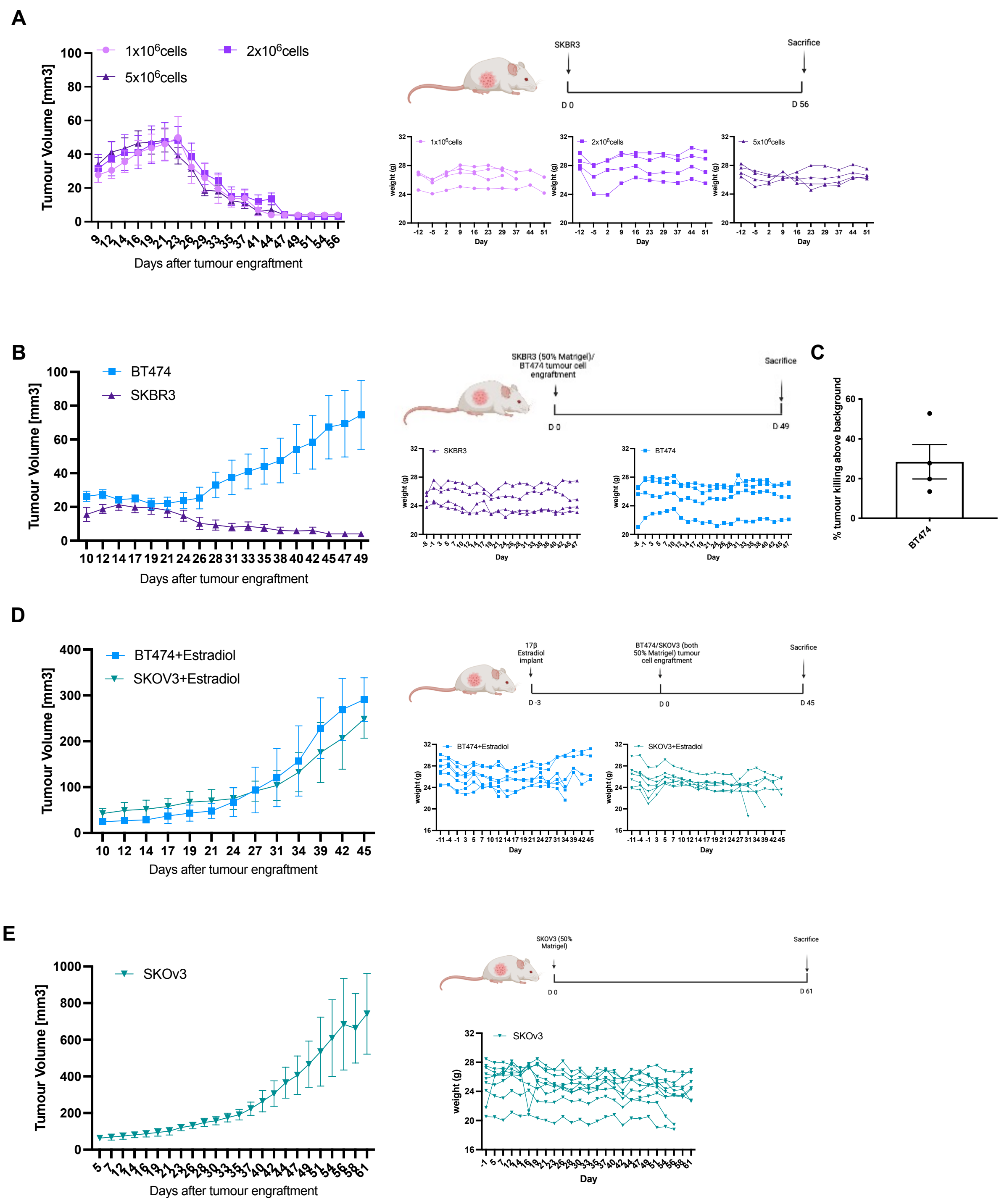

Supplementary Figure 3: High HER2 (3+) tumor model development in human CD34+ stem cell implanted mice. A) SKBR3 tumor development in CD34+ humanized mice showing tumor rejection. Left: Tumor growth curves [mm^3] following injection of SKBR3 cells at three different concentrations. Right: Weight [g] of mice through the study. B) SKBR3 and BT474 tumor development in CD34+ humanized mice showing tumor rejection (SKBR3) or slow tumor growth (BT-474). Left: Growth curves [mm^3] of SKBR3 (purple) and BT474 (blue) tumors. Right: Weight [g] of SKBR3 (purple) and

BT474 (blue) tumor-bearing mice. C) ADCC of Tras IgE with HV PBMCs against BT474 cells. D) BT474 and SKOv3 tumor development in CD34+ humanized mice with implanted 17b-estradiol pellet. Left: Growth curves [mm^3] of BT474 (blue) and SKOv3 (green) tumors. Right: Weight loss [g] of BT474 (blue) and SKOv3 (green) tumor-bearing mice observed with 17b-estradiol. C) SKOv3 tumor development in CD34+ humanized mice. Left: Growth curves [mm^3] of SKOv3 (green) tumors. Right: Weight [g] of SKOv3 (green) tumor-bearing mice.

Suppl. Figure 4

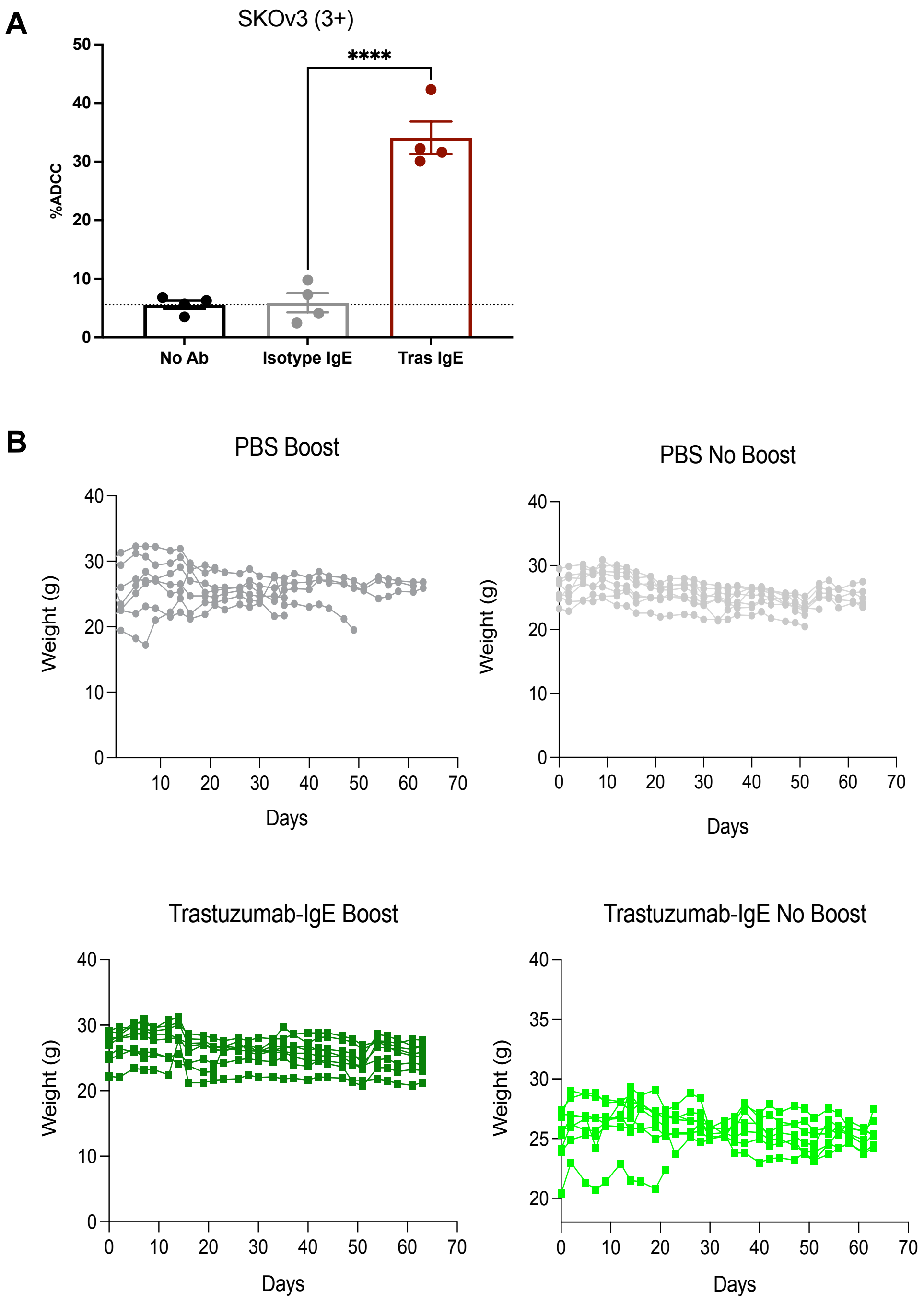

Supplementary Figure 4: SKOv3 high HER2 (3+) efficacy model and immune cell engraftment, tumor-infiltration of JIMT-1 medium HER2 (2+) efficacy model. A) Tras IgE-mediated ADCC of SKOv3 (n=4) by HV PBMCs. B) Weight [g] of treated (Tras IgE) and untreated (PBS) SKOv3 tumor-bearing mice. C) Presence of murine and human CD45^{+} cells within spleens of human PBMC engrafted HER2 2+ tumor-bearing animals (n=8). D) Immune cell infiltration of murine and human CD45^{+} cells into medium HER2+ tumors, and percentage of CD16^{+}, CD3^{+}, CD3^{-}, and NKT immune cells within

human CD45$^+$ cells (n=8). Mean of indicated independent experiments ± SEM (A) is shown. One-Way ANOVA (A); *$p \leq 0.05$; **$p \leq 0.01$; ***$p \leq 0.001$; ns non-significant.

Suppl. Figure 5

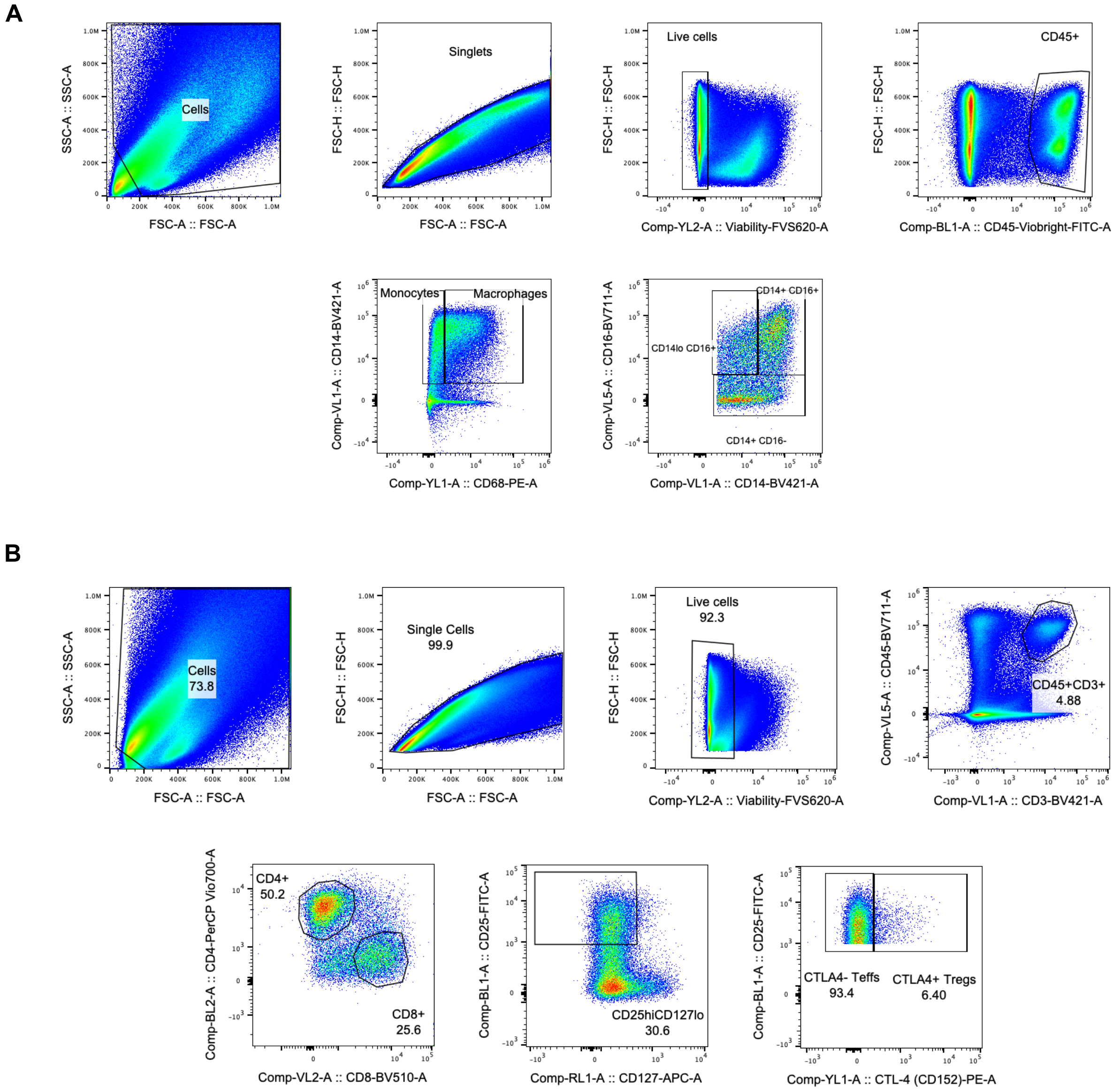

Supplementary Figure 5: Intratumoral immune cell populations in high HER2 (3+) expressing tumors. A) Gating strategy employed for evaluation of intratumoral monocytes and macrophages. B) Gating strategy employed for evaluation of intratumoral T cells.

Suppl. Figure 6

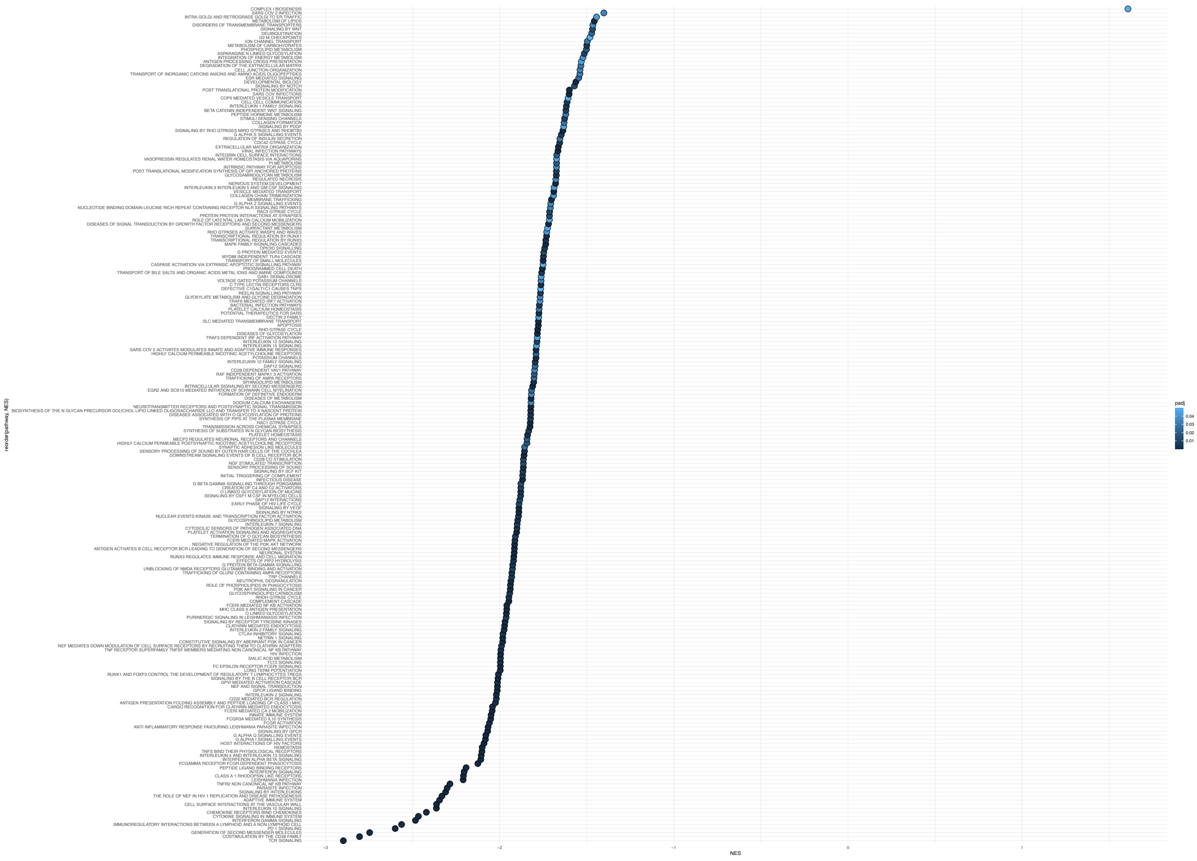

Supplementary Figure 6: Transcriptomics of medium HER2 (2+) tumors. Changes in pathways in tumors from Tras IgE treated mice (n=6) compared to untreated (n=6), as determined by gene set enrichment analysis (Reactome). All pathways with padj≤0.05 are shown.

Supplementary Methods

Cancer cell lines and quantification of HER2 expression

All cells were maintained at 5% CO_2 and 37°C. RPMI1640 and DMEM were both supplemented with 10% FCS, penicillin (5,000 U/ml), streptomycin (100 µg/ml) and will be referred to as 'complete'. Breast cancer cell lines with different HER2 expression status based on literature were sourced and cultured as follows: BT474 (HER2 3+[1], ATCC HTB-20), SKOv3 (HER2 3+[2], ATCC HTB-77), HCC1954 (HER2 3+, ATCC CRL-2338) and HCC38 (HER2 0-1+[3], ATCC CRL-2314) were cultured in complete RPMI1640, whereas SKBR3 (HER2 3+[1], ATCC HTB-30), JIMT-1 (HER2 2+,[4] DSMZ ACC 589), MDA-MB-361 (HER2 2+[4], ATCC HTB-27), MDA-MB-453 (HER2 2+[2], ATCC HTB-131), MDA-MB-175 -VII (HER2 1+[5], ATCC HTB-25) and MDA-MB-231 (HER2 0-1+[1], ATCC HTB-26) were cultured in complete DMEM. MCF-7 (HER2 0-1+[1], ATCC HTB-22) were cultured in complete DMEM supplemented with 0.01 mg/ml insulin.

Quantification of HER2 cell surface expression levels was performed using BD Quantibrite™ Beads in combination with a commercial mouse IgG2a anti-HER2 PE conjugated antibody (HRB2/258) or a non-specific isotype control. $1x10^5$ cells were incubated on ice with 0.82 ug of anti-HER2 PE or isotype control antibody for 30 min, followed by washing twice with FACS buffer (PBS pH 7.2 + 2%FBS). Sample acquisition was performed using a BD Fortessa.

Effector cells

The human monocytic cell line U937 (ATCC CRL-1593.2) and the rat basophilic cell line RBL-SX38 (transfected with the human FcεRI alpha chain; kindly provided by Prof. Jean-Pierre Kinet) were both cultured using complete RPMI, with the latter having added G418 as selection. [19]

SDS-PAGE

Samples for SDS-PAGE were prepared using BioRad 4x Sample buffer with/without 1 µl Mercaptoethanol added. Samples were heated at 95 °C for 5 min and then loaded onto a 4–20% Mini-PROTEAN® TGX™ Precast (Bio-Rad). The gel was run at constant voltage of 120 V for 1 h in 1x Tris-Glycine SDS buffer. Staining was performed using InstantBlue® Coomassie Protein Stain.

Antibody binding to HER2 antigen and Fc-receptor-expressing cells

For cell surface binding $1x10^5$ cells were incubated with trastuzumab IgE, pertuzumab IgE or isotype control at various concentrations for 30 min at 4 °C, followed by a wash with FACS buffer (PBS pH

7.2 + 2%FBS) wash. Anti-IgE FITC detection antibody was added, and cells were incubated for another 30 min at 4 °C, washed twice before acquisition.

To determine free FcεR on the surface of primary monocytes and basophils available to engage with anti-HER2 IgEs, Tras IgE and Per IgE were conjugated to AlexaFluor647 (Alexa Fluor Antibody Labelling Kit, Invitrogen). Cells were incubated with 5 µg/ml of the labelled antibodies for 30 min at 4 °C, washed with FACS buffer. Sample acquisition was performed using a BD Fortessa.

Intracellular phosphorylation assay

Phosphorylation of HER2 and total HER2 were measured using the CisBio Phospho-Her2 (Tyr1221/1222), and Total HER2 cellular Homogeneous Time Resolved Fluorescence (HTRF) kits, respectively, following manufacturer's instructions. Briefly, $5x10^4$/well cells in complete media were seeded in a 96-well flat-bottom plate and incubated overnight (5% CO_2, 37 °C). Media was replaced for 50 µl of serum-free media and incubated for another 2 hours before adding 5 µg/mL of trastuzumab IgE, pertuzumab IgE or isotype control, followed by a 30 min incubation. 50 µl of hEGF was added with a subsequent 10 min incubation. Lysing was performed using 50 µl of lysing buffer, gentle shaking for 30 min at RT. 16 µl of the lysate were transferred into a HTRF 96-well low volume plate containing 4 µl of premixed phosHer2 or Total Her2 d2 + Eu-cryptate Ab. The HTRF signal (excitation 337nm, emission 620nm (donor) and 665nm (acceptor)) was recorded after 4h incubation at room temperature.

Phosphorylation of ERK and total ERK were measured using the CisBio Phospho-ERK (Thr202/Tyr204) and Total ERK kits. The assay setup followed the same steps as described above for HER2, except that, as per manufacturer's instructions, hEGF was not included.

Viability assay

For ligand-independent cell viability assays $1x10^3$ cells/well were seeded in a clear flat-bottom 96-well plate in complete (SKBR3) or serum-reduced (1% FBS) media (SKOv3, JIMT-1) and incubated overnight at 37 °C and 5% CO_2. The following day antibodies were added at the indicated concentrations. At day 5, 100 µl of 10% MTS reagent (diluted in media) was added to the wells. After 2h of incubation at 37 °C, the plate was read at OD_{490} and OD_{650}.

Degranulation assay

RBL-SX38 cells were seeded at $1x10^4$ cells/well in a flat bottom 96 well plate in complete RPMI and incubated overnight at 37 °C, 5% CO_2. Antibodies were added at a final concentration of 1.05 nM and the plate was incubated for 1h. Afterwards the supernatant was taken off and three washes with HBSS-

1%BSA (heat shock fraction) were performed before adding $3x10^4$ target cells resuspended in HBSS-1%BSA to wells. Plates were incubated for 30 min and supernatant taken off. 20 µl of supernatant were diluted with 30 µl of HBSS-1%BSA buffer in black flat bottom 96 well plates and 50 µl of substrate (4-Methylumbelliferyl N-acetyl-β-D-glucosaminide) were added. Plates were incubated for 2 h at 37° C. The reaction was stopped by adding 100 µl of 0.5 M Tris-HCl buffer. Fluorescence was measured at 350 nm excitation and 450 nm emission using a Fluostar Omega microplate reader (BMG Labtech).

Antibody-dependent cellular cytotoxicity and phagocytosis (ADCC/ADCP) assay

ADCC/ADCP assays were performed according to Bracher et al.,2007[6]. Briefly, target cells were stained with CFSE (CarboxyFluoroscein Succinimidyl Ester) the day prior to the experiment. The next day freshly isolated PBMCs from healthy volunteers or U937 (effector cells) were added at E:T 10:1 or 2:1, respectively, to the target cells in the presence of 5 ug/ml of antibody. A no antibody control served as baseline. Samples were incubated for 3h at 37°C and 5%CO_2. Supernatants were taken off and cells were washed with FACS buffer prior to staining with anti-CD45-APC (PBMCs) or anti-CD89 APC (U937s) for 30 min at 4 °C. Samples were washed again and DAPI was added to the samples prior to acquisition using a BD Fortessa. The following controls were used for compensation: 1. Unstained tumor cells mixed with unstained effector cells, 2. anti-CD45-APC/anti-CD89-APC-stained effector cells, 3. CFSE-stained tumor cells and 4. killed unstained tumor cells with DAPI. Analysis was performed using FlowJo. ADCC and ADCP were calculated according to the formula below.

$$ADCC = 100 * \left[\frac{((Average\ R1_{No\ Ab} - R1_x) + R3_x)}{Average\ R1_{\ No\ Ab}}\right]$$

$$ADCC = 100 * \left[\frac{((Average\ tumour\ cells\ in\ untreated\ (No\ Ab) sample - tumour\ cells\ in\ treated\ sample) + Dead\ tumour\ cells)}{Average\ tumour\ cells\ in\ untreated\ (No\ Ab) sample}\right]$$

$$ADCP = 100 * \left[\frac{R2_x}{Average\ R1_{No\ Ab}}\right]$$

$$ADCP = 100 * \left[\frac{(Phagocytosed\ tumour\ cells)}{Average\ tumour\ cells\ in\ untreated\ (No\ Ab) sample}\right]$$

R1 = total CFSE+ cell count. R2= CFSE+APC+ cell count. R3= CFSE+DAPI+ cell count

Immune effector cell crosslinking assay

Primary monocytes (1×10^6 cells/mL) were incubated with 5 µg/mL IgE, or complete RPMI, for 1 hour at 37°C, 5% CO_2. Following washing, 10 µg/mL polyclonal goat anti-human IgE (Abcam) were added

to induce crosslinking and incubated for 1 h at 37°C, 5% CO_2 at 37°C. Cells were washed, re-suspended in complete RPMI and incubated for 24 h at 37°C, 5% CO_2. Levels of IL-1β, IL-6, IL-10, TNF-α (Mabtech) and CCL-2/MCP-1 (Invitrogen) in cell culture supernatants were measured by respective ELISAs. Plates were read using a Flurostar Omega Spectrophotometer (BMG Labtech).

Evaluation of cell surface marker expression of stimulated monocytes was performed by flow cytometry using Fc receptor Block (BD Biosciences), viability DAPI stain (BioLegend), anti-CD14-BUV395 (clone: MøP9); BD Biosciences), anti- CD40-BV421 (clone: 5C3; BioLegend), anti-CD80-PE (clone: 2D10; BioLegend), anti-CD86- BUV737 (clone: 2331 (FUN-1); BD Biosciences), anti-CD163-APC (clone: GHI/61; BioLegend), anti-CCR2-BV605 (clone: K036C2; BioLegend), anti-PDL1-FITC (clone: MIH3; BioLegend) and anti-HLA-DR-Alexa Fluor 700 (clone: L243, BioLegend). Monocytes were incubated with Fc block for 10 min at room temperature, washed and incubated with the appropriate antibodies for 30 min at 4 °C. Cells were washed and 10^7 viable monocytes were acquired.

Cytokine analysis of co-cultures

Supernatants of co-cultures were obtained from ADCC/ADCP set ups (as described above). To assess levels of TNF-α, IL-6 and CCL2/MCP-1 in these undiluted supernatants a Luminex assay (BioTechne) was performed as per manufacture's protocol and acquired using the Luminex FlexMap 3D.

Basophil Activation Test (BAT) assay

Basophil Activation Tests (BAT, Flow2 CAST® kit, Bühlmann Laboratories AG, Schönenbuch, Switzerland) were performed, according to the manufacturer's instructions and as previously described, within 4h after blood collection[7,8].

In vivo model development and antibody treatment efficacy studies

All procedures were performed under aseptic conditions. Mice were kept under pathogen-free conditions on a 12-hour light/dark cycle (light of 350–400 lux). Housing conditions were maintained at 22 °C, and at a relative humidity of 40 to 60%. For all studies animals were sacrificed for ethical reasons if required due to tumor condition (e.g. necrosis), body weight loss (>20%) or clinical score.

HER2 3+ trastuzumab-resistant model: All *in vivo* studies performed used highly immunodeficient female mice (NOD-Prkdcem26Cd52Il2rgemCd22) engrafted with human hematopoietic Stem cells (CD34+). Tumor volume and clinical health scores were monitored 3 times per week using caliper. Tumor volume was calculated using the formula: (Length x (Width)2 /2).

Model development:

SKBR3 cancer cell implantation: Female humanized NCG mice were randomized into three groups of four mice according to their humanization rate and CD34+ cell donors. The three groups were engrafted subcutaneously on the right flank with 1, 2 or $5x10^6$ of SKBR3 tumor cells respectively.

SKBR3 and BT474: Female humanized NCG mice were randomized and injected *s.c.* with $5x10^6$ SKBR3 (in 50% Matrigel) or BT474 tumor cells per mouse (four per group).

BT474 and SKOv3 cell implantation with estrogen: Female humanized mice were supplemented i*n vivo* with a pellet of 17β-Estradiol (0,18 mg, 60 days of release time, Innovative Research of America) 3 days prior to tumor cell engraftment (D-3). Mice were randomized and seven mice per group were injected *s.c.* with $5x10^6$ BT474 or SKOv3 tumor cells, in 50% Matrigel.

SKOv3 cancer cell implantation: Female humanized mice (n=9) were injected *s.c.* with $5x10^6$ SKOV-3 tumor cells per mouse.

Tras IgE efficacy studies:

SKOv3 In vivo efficacy study: On Day 0 (D0) $5x10^6$ SKOV-3 tumor cells were subcutaneously injected into 16 female humanized NCG mice. When tumor volume reached around 40/80 mm^3 at D14, mice were randomized and injected twice per week until D54 with PBS or Tras IgE at 20 mg/kg (n=8 per group). After treatment stop, mice were monitored for a further 10 days before sacrifice (D64). Body weight, clinical score and tumor volume were monitored 2 to 3 times per week.

HER2 2+ Trastuzumab-resistant mode efficacy study:

JIMT-1 cells were implanted at 1 x 10^7 in matrigel on the rear dorsum of female NXG mice (Janvier Labs, France). Tumors were measured three times a week, starting 7 days after implantation. When tumors were approximately 50mm^3, mice were engrafted human PBMCs from three donors via the lateral tail vein (8 mice per PBMC donor). PBMC engraftment was confirmed by flow cytometry. Dosing of PBS or Tras IgE at 20 mg/kg twice a week (n=12 per group, randomized) was started together with PBMC engraftment. Animals were sacrificed on Day 42, tumors excised and frozen in RNALater.

Power calculation to determine sample sizes (5% significance, 90% statistical power) are based on a two-tailed, Mann Whitney U test. Stratified randomization has been performed for all efficacy studies.

An independent researcher was assigned to blindly measure parameters such as tumor size to reduce or eliminate some sources of experimental bias.

Flow cytometry analyses of SKOv3 tumor-associated immune infiltrates

In the above described *in vivo SKOv3 efficacy* study, tumors were harvested at the time of euthanasia, and enzymatically dissociated. Only tumors from animals sacrificed at day 63 (end of study) were included for the assessment of tumor-associated immune infiltrates. Cells were stained for immunophenotyping according to panel 1 or 2 (see below). Cells were incubated for 5 min at 4°C with FcR blocking solution (Miltenyi, 130-059-901) followed by an incubation of 15 min at 4°C with Live/Dead FVS 620. Subsequently cells were stained with the respective antibodies and incubated for 30 min at 4°C. For panel 2 fixation/permeabilization was additionally performed (20min at 4°C) followed by intracellular staining (30 min at 4°C) before acquisition. Flow cytometry was performed on an Attune NxT Flow Cytometer (Life Technologies).

Panel 1 (T cell panel): anti-CD3-BV421 (Clone:SK7,BioLegend), anti-CD8-BV510 (Clone SK1, BD Biosciences), anti-CD45-BV711 (Clone HI30, BD Biosciences), anti-CD25 FITC (Clone 4E3, Miltenyi), anti-CD4 PerCP Vio700(Clone VIT4, Miltenyi), anti- CTLA-4 PE (Clone REA1003, Miltenyi), Viability FVS620 (BD Biosciences), anti-PD1 PE-Cy7 (Clone EH12 2H7, BioLegend) and anti-CD127 APC (Clone MB15-18C9, Miltenyi)

Panel 2 (Myeloid panel): anti-CD14 BV421 (Clone M5E2, BD Biosciences), anti FcERI BV510 (Clone AER-37, BioLegend), anti-CD86 BV650 (Clone 2331, BD Biosciences), anti-CD16 BV711 (Clone 3G8, BD Biosciences), anti-CD80 BV786 (Clone 2D10, Biolegend), anti-CD45 viobright-FITC (Clone 5B1, Miltenyi), anti-CD23 BB700 (Clone M-L233, BD Biosciences), anti-CD68 PE (Clone Y1/82A, Biolegend), Viability FVS620 (BD Biosciences), anti-PDL-1 PE-Cy7 (Clone 29E.2A3, Miltenyi), anti-CD163 APC (Clone REA812, Miltenyi) and anti-CD206 APC-Cy7 (Clone 43876, Biolegend).

Bulk RNA sequencing and transcriptomic analysis of JIMT-1 tumors

For bulk RNA sequencing a total of 6 tumors (2 per donor) per treatment group were selected and sent for RNA extraction and bulk RNA sequencing to Azenta/Genewiz. Differentially expressed genes between treatment and control tumors were identified using the package DESeq2 (1.40.2). For gene overrepresentation analysis (GOA), data was divided into the top 200 up- and downregulated genes, respectively, according to log2 fold change and padj ≤ 0.05 and analyzed using gprofiler2 (0.2.2). For gene set enrichment analysis (GSEA) all genes, ranked according to fold change, were used to calculate enrichment of gene sets within Reactome (7.4) using package fgsea (1.26.0) with random seed set to 2. Deconvolution was performed using TPM-normalized data and the ConsensusTME (0.0.1.9000) package with cancer type defined as 'BRCA' and Single sample GSEA (ssGSEA) chosen as statistical method. R version 4.3.1 (2023-06-16) was used.

Statistical analyses

Data are presented as mean ± Standard Deviation (SD) or Standard Error of the Mean (SEM) as indicated in the figure legends. GraphPad Prism was used to perform statistical analyses. A Shapiro–Wilk normality test was performed to evaluate normality. Subsequent appropriate statistical analyses were performed (two-tailed unpaired Student's t test, One-way ANOVA for normally distributed data; Mann-Whitney or Kruskal-Wallis test for non-parametric data and two-way ANOVA), and statistically significant differences are indicated in the graphs or additional tables were indicated. p values: *$p \leq 0.05$; **$p \leq 0.01$; ***$p \leq 0.001$; ****$p \leq 0.0001$.

References

1. Subik, K. *et al.* The Expression Patterns of ER, PR, HER2, CK5/6, EGFR, Ki-67 and AR by Immunohistochemical Analysis in Breast Cancer Cell Lines. *Breast Cancer (Auckl)* **4**, 35 (2010).
2. Saigo, K. *et al.* The use of cell line standards to reduce HER-2/neu assay variation in multiple European cancer centers and the potential of automated image analysis to provide for more accurate cut points for predicting clinical response to trastuzumab. *Am J Clin Pathol* **122**, 51–55 (2004).
3. Li, Y. *et al.* Targeted immunotherapy forHER2-low breast cancer with 17p loss. *Sci Transl Med* **13**, (2021).
4. Li, J. Y. *et al.* A Biparatopic HER2-Targeting Antibody-Drug Conjugate Induces Tumor Regression in Primary Models Refractory to or Ineligible for HER2-Targeted Therapy. *Cancer Cell* **29**, 117–129 (2016).
5. Dako. *HercepTest TM Interpretation Manual Breast Cancer EDUCATION.*
6. Bracher, M., Gould, H. J., Sutton, B. J., Dombrowicz, D. & Karagiannis, S. N. Three-colour flow cytometric method to measure antibody-dependent tumour cell killing by cytotoxicity and phagocytosis. *J Immunol Methods* **323**, 160–171 (2007).
7. Bax, H. J. *et al.* Basophils from Cancer Patients Respond to Immune Stimuli and Predict Clinical Outcome. **9**, (2020).
8. Bax, H. J. *et al.* Basophil activation test in cancer patient blood evaluating potential hypersensitivity to an anti-tumor IgE therapeutic candidate. *Allergy* **75**, (2020).
